# Guest Ion‐Dependent Reaction Mechanisms of New Pseudocapacitive Mg_3_V_4_(PO_4_)_6_/Carbon Composite as Negative Electrode for Monovalent‐Ion Batteries

**DOI:** 10.1002/advs.202207283

**Published:** 2023-02-15

**Authors:** Qiang Fu, Björn Schwarz, Ziming Ding, Angelina Sarapulova, Peter G. Weidler, Alexander Missyul, Martin Etter, Edmund Welter, Weibo Hua, Michael Knapp, Sonia Dsoke, Helmut Ehrenberg

**Affiliations:** ^1^ Institute for Applied Materials (IAM) Karlsruhe Institute of Technology (KIT) Hermann‐von‐Helmholtz‐Platz 1 D‐76344 Eggenstein‐Leopoldshafen Germany; ^2^ Institute of Nanotechnology (INT) Karlsruhe Institute of Technology (KIT) Hermannvon, Helmholtz‐Platz 1 D‐76344 Eggenstein‐Leopoldshafen Germany; ^3^ Technische Universität Darmstadt 64289 Darmstadt Germany; ^4^ Institute of Functional Interfaces (IFG) Chemistry of Oxidic and Organic Interfaces (COOI) Karlsruhe Institute of Technology (KIT) Hermann‐von‐Helmholtz‐Platz 1 D‐76344 Eggenstein‐Leopoldshafen Germany; ^5^ CELLS‐ALBA Synchrotron Cerdanyola del Valles Barcelona E‐08290 Spain; ^6^ Deutsches Elektronen‐Synchrotron (DESY) Notkestr. 85 22607 Hamburg Germany; ^7^ School of Chemical Engineering and Technology Xi'an Jiaotong University Xi'an Shaanxi 710049 P. R. China

**Keywords:** in operando synchrotron diffraction, in operando X‐ray absorption spectroscopy, magnetic properties, monovalent‐ion batteries, triclinic Mg_3_V_4_(PO_4_)_6_

## Abstract

Polyanion‐type phosphate materials, such as M_3_V_2_(PO_4_)_3_ (M = Li/Na/K), are promising as insertion‐type negative electrodes for monovalent‐ion batteries including Li/Na/K‐ion batteries (lithium‐ion batteries (LIBs), sodium‐ion batteries (SIBs), and potassium‐ion batteries (PIBs)) with fast charging/discharging and distinct redox peaks. However, it remains a great challenge to understand the reaction mechanism of materials upon monovalent‐ion insertion. Here, triclinic Mg_3_V_4_(PO_4_)_6_/carbon composite (MgVP/C) with high thermal stability is synthesized via ball‐milling and carbon‐thermal reduction method and applied as a pseudocapacitive negative electrode in LIBs, SIBs, and PIBs. In operando and ex situ studies demonstrate the guest ion‐dependent reaction mechanisms of MgVP/C upon monovalent‐ion storage due to different sizes. MgVP/C undergoes an indirect conversion reaction to form Mg^0^, V^0^, and Li_3_PO_4_ in LIBs, while in SIBs/PIBs the material only experiences a solid solution with the reduction of V^3+^ to V^2+^. Moreover, in LIBs, MgVP/C delivers initial lithiation/delithiation capacities of 961/607 mAh g^−1^ (30/19 Li^+^ ions) for the first cycle, despite its low initial Coulombic efficiency, fast capacity decay for the first 200 cycles, and limited reversible insertion/deinsertion of 2 Na^+^/K^+^ ions in SIBs/PIBs. This work reveals a new pseudocapacitive material and provides an advanced understanding of polyanion phosphate negative material for monovalent‐ion batteries with guest ion‐dependent energy storage mechanisms.

## Graphical abstract

Mg_3_V_4_(PO_4_)_6_/carbon composite shows large electrochemical difference for Li^+^ and Na^+^/K^+^ storage suggests different electrochemical mechanisms among Li^+^ and Na^+^/K^+^ storage. In operando studies demonstrate that Mg_3_V_4_(PO_4_)_6_/carbon undergoes an indirect conversion reaction for Li^+^ storage, while the material experiences a solid solution for Na^+^/K^+^ storage. This work reveals a new pseudocapacitive material Mg_3_V_4_(PO_4_)_6_/C and guest ion‐dependent energy storage mechanisms of Mg_3_V_4_(PO_4_)_6_/C.

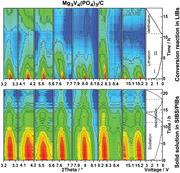

## 1 Introduction

Currently, monovalent‐ion batteries, in particular, lithium‐ion batteries (LIBs) are playing critical roles in modern society and our daily life and have achieved great success in applications for portable electrical devices and electronic cars^[^ ^1^ ^]^ due to their high energy densities, lightweight, and long cycle life.^[^ ^2^ ^]^ Meanwhile, some drawbacks need to be overcome, such as the limited capacities of the commercial negative electrode materials, the high cost of lithium, and potential safety concerns.^[^ ^1^, ^2^ ^]^ As monovalent‐ion batteries, sodium‐ion batteries (SIBs), and potassium‐ion batteries (PIBs), which share a similar “rocking chair” working principle to LIBs, have received much attention and are considered as promising candidates for large‐scale energy storage, thanks to their abundant resources and low cost.^[^ ^3^ ^]^ Moreover, aluminum can be used as the current collector in positive and negative electrodes for both SIBs and PIBs, whereas an expensive and heavier Cu foil must be used in negative electrodes of LIBs, due to the Li–Al alloy formation at low potential.^[^ ^4^ ^]^ The potassium‐ion in propylene carbonate (PC) displays a lower ion‐solvent interaction due to lower desolvation energy and a smaller solvated ion as compared to Li‐ and Na‐ions, resulting in fast diffusion kinetics and high rate capability.^[^ ^5^ ^]^ Many different types of cathode and anode materials have been studied for monovalent‐ion storage so far, including transition metal oxides, Prussian blue analogs, polyanionic compounds, fluorides, organic materials, sulfur‐based materials, titanium‐based, alloy based and carbonaceous materials.^[^ ^1^, ^6^ ^]^ However, in SIBs and PIBs, big challenges still exist in developing electrode materials suitable for commercialization due to the large ionic radius of Na^+^ (1.02 Å) and K^+^ (1.38 Å)^[^ ^2d^ ^]^ resulting in sluggish diffusion kinetics. Moreover, safety concerns and the reactive property of Na and K metal make them very difficult as direct negative electrodes for commercial cells.

The famous graphite negative electrode demonstrates guest‐dependent reaction mechanisms upon monovalent‐ion insertion with the formation of LiC_6_, NaC_64_, and KC_8_, respectively. Nowadays, graphite is commonly used as negative electrode material for LIBs because of its flat and low working potential (0.1–0.2 V vs Li^+^/Li) and relatively high specific capacity (372 mAh g^−1^).^[^ ^7^ ^]^ Despite its high specific capacity, the very low operating voltage close to lithium plating may lead to safety concerns.^[^ ^8^ ^]^ Moreover, graphite is electrochemically less active with the formation of NaC_64_ in Na cells and cannot be used as an insertion host of SIBs since Na^+^ insertion into graphite is significantly impeded.^[^ ^9^ ^]^ This might be due to the unfavorable mismatch between the graphite structure and the size of the Na ion and the energetic instability of the Na‐graphite intercalation compounds (GICs).^[^ ^9b^ ^]^ Surprisingly, graphite can form KC_8_ by K^+^ ion insertion with a high initial charge capacity of 273 mAh g^−1^ in PIBs. However, KC_8_ suffers from large volume expansion during potassium ions intercalation/deintercalation, which might cause safety issues and low coulombic efficiency.^[^ ^10^ ^]^ Despite the good Na storage performance in hard carbon, the likely formation of sodium dendrites at a low working voltage (below 0.1 V) can cause safety issues.^[^ ^11^ ^]^

Recently, polyanion‐type phosphate materials have gripped much attention due to their pronounced structural and thermal stability and open framework.^[^ ^12^ ^]^ Among them, vanadium‐based polyanion‐type compounds are very promising. For example, both monoclinic and rhombohedral Li_3_V_2_(PO_4_)_3_ (LVP) can be utilized as both positive and negative materials in LIBs due to the variety of oxidation states of vanadium and its open structure.^[^ ^13^ ^]^ The monoclinic LVP negative electrode exhibits a stable reversible capacity of 203 mAh g^−1^ with multiple plateaus in the potential ranges of 3.0–0.0 V versus Li^+^/Li as reported by Rui et al.^[^ ^14^ ^]^ while the rhombohedral LVP shows a reversible capacity of ≈115 mAh g^−1^ with a plateau at ≈1.75 V in the potential range of 3.0–1.0 V versus Li^+^/Li as reported by Jian et al.^[^ ^15^ ^]^ Fu et al.^[^ ^16^ ^]^ studied calcium‐substituted monoclinic LVP negative electrodes in LIBs, all of which showed improved electrochemical performance and can deliver specific capacities higher than 50 mAh g^−1^ at the extremely high C‐rate of 200 C. LVP/C undergoes two‐phase reaction during cycling, whereas Ca_1.5_V_2_(PO_4_)_3_/C shows capacitive‐like mechanism. NASICON‐structured Na_3_V_2_(PO_4_)_3_ (NVP) can serve as both positive and negative electrode material for SIBs, because of its unique structural feature and multi redox couples of V. Jian et al.^[^ ^17^ ^]^ first reported NVP as a negative electrode for SIBs by combined computational and experimental studies. The NVP material exhibits two pairs of redox peaks at around 1.6 and 0.3 V versus Na^+^/Na with a reversible charge capacity of ≈150 mA h g^−1^, exhibiting two‐phase reaction during cycling.^[^ ^18^ ^]^ The symmetric Na‐ion full cell consisting of Na_2_VTi(PO_4_)_3_/C reported by Wang et al.^[^ ^19^ ^]^ exhibited a superior rate capability and long lifetime over 10 000 cycles, where Na_2_VTi(PO_4_)_3_/C shows three plateaus at 3.4, 2.1, and 1.6 V (V^4+^/V^3+^, Ti^4+^/Ti^3+^, and V^3+^/V^2+^ redox couples, respectively) in the potential range of 1.5–4.5 V versus Na^+^/Na. NASICON‐type K_3_V_2_(PO_4_)_3_ (KVP) delivers a reversible specific capacity of 130 and 91 mAh g^−1^ with the formation of K_5_V_2_(PO_4_)_3_ and KV_2_(PO_4_)_3_ as both negative and positive electrodes in PIBs, respectively, in the range of 1.0–2.5 and 2.0–4.0 V versus K^+^/K.^[^ ^20^ ^]^ Interestingly, the abovementioned M_3_V_2_(PO_4_)_3_ (M = Li/Na/K) only serve as insertion‐type hosts and exhibit clear redox peaks, typical for battery‐type materials (with diffusion‐controlled kinetics). Nevertheless, it remains a great challenging to understand the reaction mechanism of materials upon monovalent‐ion insertion. Magnesium vanadium phosphate Mg_3_V_4_(PO_4_)_6_ (MgVP) crystallizes in the space group $P\bar{1}$ and is isostructural with a known quaternary V^3+^‐containing Zn_3_V_4_(PO_4_)_6_^[^ ^21^ ^]^ which has been prepared recently and whose magnetic property is once evaluated. It consists of bioctahedral V_2_O_10_ units of two edge‐sharing octahedra, monophosphate groups, distorted MgO_6_ octahedra, and distorted MgO_5_ trigonal bipyramids. Its structure suggests that MgVP can be a promising negative electrode material with high thermal stability. However, its electrochemical properties and reaction mechanisms in Li/Na/K‐ion batteries have not been studied so far.

Here in this work, we report the synthesis of MgVP/C composite via ball‐milling and the carbon‐thermal reduction method, where in situ formed carbon not only could relieve the growing up and aggregation of particles during annealing, but also increase the electronic conductivity of the electrode. The electrochemical performance of MgVP/C in LIBs, SIBs, and PIBs was studied, demonstrating significant differences in terms of electrochemical reaction among Li^+^ and Na^+^/K^+^. The guest ion‐dependent electrochemical reaction mechanisms upon Li‐, Na‐, and K‐ion insertion were carefully investigated via in operando synchrotron diffraction and X‐ray absorption spectroscopy (XAS) together with ex situ magnetic measurements.

## 2 Result and Discussion

### 2.1 Structural and Morphological Characterization

**Figure 1:**
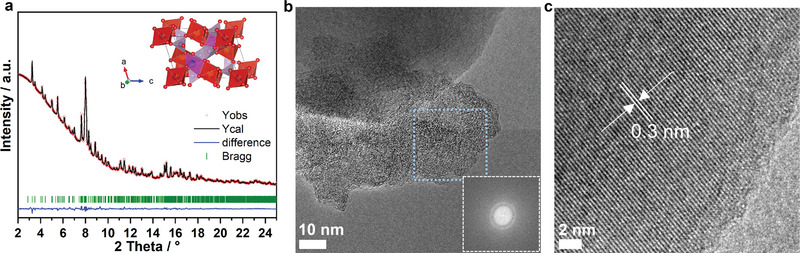
Crystal structure of the pristine MgVP/C. Rietveld refinement against synchrotron diffraction data (*λ* = 0.41273 Å converted from *λ* = 0.20723 Å, where the data were collected at P02.1, DESY) a) and HRTEM image of MgVP/C b,c) (inset is the FFT pattern of a selected region).

The crystal structure of the as‐prepared sample was characterized by synchrotron diffraction, as shown in **Figure** **1**a. All featured reflections observed for the pristine MgVP/C can be well indexed according to triclinic Mg_3_V_4_(PO_4_)_6_ with a space group of $P\bar{1}$, in agreement with a previous report.^[^ ^22^ ^]^ The Rietveld refinement result demonstrates that its lattice parameters are *a* = 6.325(1) Å, *b* = 7.903(1) Å, *c* = 9.286(1) Å, *α* = 105.287(3)°, *β* = 108.567(3)°, and *γ* = 101.339(3)° (Table S1, Supporting Information). Moreover, there is no evidence for diffraction reflections belonging to the graphitic carbon, suggesting an amorphous state of carbon. Hence, MgVP/C composite is successfully prepared. To estimate the amount of carbon in MgVP/C, TGA was performed under O_2_ flow. Figure S1a (Supporting Information) demonstrates that the slight weight loss below 300 °C is attributed to the evaporation of absorbed and chemically bonded water in the sample. With further heating, an abrupt weight loss is observed between 300 and 500 °C and is ascribed to the combustion of residual carbon. Therefore, the amount of carbon in MgVP/C material is determined as 4.9 wt%. Transmission electron microscopy (TEM) (Figure S2, Supporting Information) reveals that MgVP/C consists of aggregated submicron particles as well as smaller aggregated carbon particles in between. The microstructure of MgVP/C was investigated using high‐resolution TEM (HRTEM) as displayed in Figure 1b,c and Figure S2b,c in the Supporting Information, indicating that MgVP is highly crystalline, as well as amorphous carbon was distributed unevenly between the aggregated particles as a connection between MgVP crystalline grains. The fast Fourier transform (FFT) analysis of the HRTEM image confirms the amorphous features of carbon, in agreement with the synchrotron radiation diffraction result. The BET surface area of Mg_3_V_4_(PO_4_)_6_/C is 78 m^2^ g^−1^, which is much larger than that of LVP/C (54.3 m^2^ g^−1^) and calcium substituted LVP (25–40 m^2^ g^−1^)^[^ ^16^ ^]^ and the pore‐size distribution curves show that the pore size lies in both, the micro‐ and mesopore range (Figure S1b, Supporting Information). This can be beneficial to the electrochemical reaction and kinetics due to good electrolyte penetration and short diffusion distances.^[^ ^23^ ^]^

### 2.2 Electrochemical Properties

The electrochemical activity of MgVP/C negative electrode in LIBs, SIBs, and PIBs was studied by galvanostatic charge‐discharge cycling in the potential range of 3.0–0.01 V versus Li^+^/Li, Na^+^/Na, and K^+^/K, respectively, at 50 mA g^−1^ at 25 °C.

**Figure 2:**
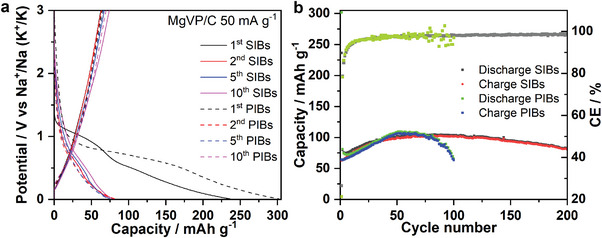
Na and K insertion performance in MgVP/C. The discharge–charge curves a) and cycling performance b) of MgVP/C at 50 mA g^−1^ at 25 °C for both SIBs and PIBs, respectively.

As shown in **Figure** **2**a, MgVP/C displays two slope‐like plateaus at 1.0 and 0.5 V during the 1st sodiation process (discharge). Afterward, only a slope‐like curve can be observed during the 1st desodiation process and the following cycles in SIBs. MgVP/C delivers initial sodiation and desodiation capacities of 236 and 63 mAh g^−1^, respectively, resulting in a very low Coulombic efficiency (CE) of 27% with an initial irreversible capacity loss of 173 mAh g^−1^. In the 2nd cycle, the sodiation and desodiation capacities are 80 and 63 mAh g^−1^, respectively, giving a reduced irreversible capacity loss (17 mAh g^−1^). After the 2nd cycle, MgVP/C displays a capacity increase to 104 mAh g^−1^ at the 64th cycle and is around 81 mAh g^−1^ after 200 cycles (Figure 2b). Compared with that in SIBs, MgVP/C in PIBs only exhibits one slope‐like plateau at 0.75 V during the 1st potassiation process and similar slope‐like curves during the 1st depotassiation process and the following cycles. The MgVP/C electrode shows initial potassiation and depotassiation capacities of 301 and 64 mAh g^−1^, respectively, with a low CE of 21% and an initial irreversible capacity loss of 237 mAh g^−1^. Similarly, the potassiation capacity quickly drops to 81 mAh g^−1^ for the 2nd cycle and increases to 109 mAh g^−1^ at the 51st cycle, and finally offers a capacity of 66 mAh g^−1^ after 100 cycles (Figure 2b). The origin of fast capacity decay after 50 cycles in PIBs could be attributed to material degradation, electrolyte consumption, and side reactions, where the electrolyte is not optimized in a half cell.^[^ ^24^ ^]^ However, the detailed reasons behind this need to be elucidated in future work.

**Figure 3:**
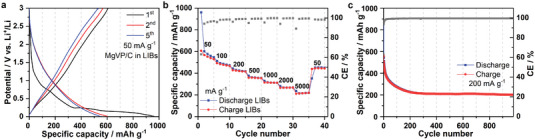
Electrochemical performance of MgVP/C in LIBs. The discharge–charge curves at 50 mA g^−1^ a), rate capability b), and cycling performance at 200 mA g^−1^ c) of MgVP/C at 25 °C for LIBs.

Compared to SIBs and PIBs, MgVP/C in LIBs (**Figure** **3**a) displays one slope‐like plateau at 0.7 V and one very long plateau at 0.15 V during the 1st lithiation process (discharge). MgVP/C delivers high initial lithiation and delithiation capacities of 961 and 607 mAh g^−1^, respectively, resulting in an initial irreversible capacity loss of 354 mAh g^−1^. It shows CE of 63% for the first cycle in LIBs, which is much larger than that in SIBs and PIBs. Those large irreversible capacity losses could be assigned to the formation of a thick solid electrolyte interface (SEI) layer and the decomposition of the electrolytes, as well as to both the amorphous carbon and C65 conductive additive in the Li/Na/K‐ion system at low potential.^[^ ^14^, ^25^ ^]^ Strategies including electrolyte engineering, structure design, morphology tuning, defects engineering, and binder optimization are effective methods to improve the initial CE of electrode materials.^[^ ^26^ ^]^

To estimate the capacity contribution of C65, C65 electrodes were prepared using C65 and PTFE binder in a ratio of 8:2 because of the difficulty of electrode coating with PVDF. The C65 electrode shows clear plateaus at 0.72, 0.90, and 0.80 V for the initial lithiation, sodiation, and potassiation process, respectively, and only slope‐like profiles afterward (Figure S3a, Supporting Information). The C65 electrode delivers initial discharge/charge capacities of 385/198, 339/77, and 390/124 mAh g^−1^ at 50 mA g^−1^ and a second discharge capacity of 232, 93, and 162 mAh g^−1^ with a capacity retention of 98%, 83%, and 73% (in term of the second discharge capacity) for LIBs, SIBs, and PIBs, respectively (Figure S3b, Supporting Information). The formation of SEI also contributes to the capacity, especially for the first discharge process. Therefore, C65 in the MgVP/C electrode roughly provides additional discharge/charge capacities of 77/40 (corresponding to 2.4/1.3 Li^+^ ions), 68/15, and 78/25 mAh g^−1^ for the first cycle in LIBs, SIBs, and PIBs, respectively. The material shows lithiation and delithiation capacities of 600 and 567 mAh g^−1^ at the second cycle, respectively, giving a reduced irreversible capacity loss (33 mAh g^−1^). Different from SIBs and PIBs, the MgVP/C in LIBs does not exhibit an activation and its lithiation capacity quickly degrades to 547 mAh g^−1^ at the 5th cycle. Note that the large first and second lithiation capacities of 961 and 600 mAh g^−1^ correspond to around 30 and 19 Li^+^ ions per MgVP/C, respectively. Therefore, MgVP/C must undergo a conversion reaction to obtain such high capacity; for example, 18 Li^+^ ions to one Mg_3_V_4_(PO_4_)_6_ is needed to form the final product of Mg^0^, V^0^, and Li_3_PO_4_ via the following equation (1). Whereas it is still not clear whether the material goes through a direct conversion reaction (1) or it starts with an insertion reaction followed by a conversion reaction

(1) $${\mathrm{Mg}}_{3}V_{4}{\left({\mathrm{PO}}_{4}\right)}_{6}+18{\mathrm{Li}}^{+}+18e^{-}\to 3{\mathrm{Mg}}^{0}+4V^{0}+6{\mathrm{Li}}_{3}{\mathrm{PO}}_{4}$$

The rate capability of MgVP/C was investigated by applying currents ranging from 50 mA g^−1^ to 5000 mA g^−1^, as displayed in Figure 3b. MgVP/C provides high specific lithiation capacities of 485 and 424 mAh g^−1^ at 100 and 200 mA g^−1^, respectively. Surprisingly, the material can still deliver a high lithiation capacity of 216 mAh g^−1^ with further increasing the current density to 5000 mA g^−1^. A high capacity retention of 39% is achieved when the current density increases by 100 times from 50 to 5000 mA g^−1^, indicating fast Li^+^ migration in the MgVP/C host. With the high current of 200 mA g^−1^, the material shows an initial lithiation/delithiation capacity of 882/557 mAh g^−1^ and undergoes a rapid capacity decay to 226 mAh g^−1^ during the first 200 cycles and stabilizes at around 200 mAh g^−1^ after 980 cycles (Figure 3c). The high initial reversible capacity of MgVP/C is much higher than that of graphite (372 mAh g^−1^),^[^ ^7^ ^]^ indicating its promising potential in practical application. Although its cycling stability is not as good as that of graphite, further efforts such as nanoengineering and surface coating of MgVP/C, and electrolyte engineering could be effective approaches to improve the reversibility and the electrochemical performance of MgVP/C.

### 2.3 Reaction Mechanisms

**Figure 4:**
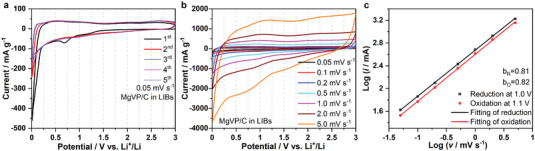
Kinetics properties of MgVP/C in LIBs. The CV curves of MgVP/C at the scan rate of 0.05 mV s^−1^ a), third CV curves at different scan rates from 0.05 to 5.0 mV s^−1^ b) in the potential range of 3.0–0.01 V versus Li^+^/Li, and linear fitting of the log(*i*) versus log(*v*) plots c) based on CV curves in (b).

The working mechanism of MgVP/C was studied by combining electrochemical and spectroscopic techniques. **Figure** **4**a displays the cyclic voltammetry (CV) of MgVP/C at scan rates of 0.05 mV s^−1^ for LIBs. A broad reduction peak at 0.7 V and a peak near 0 V can be seen in the first cycle. The peak at 0.7 V is assigned to the SEI formation and does not appear in the following cycles. While the peak near 0.1 V can be ascribed to the Li^+^ ions insertion into amorphous carbon and the SEI formation due to the decomposition of the electrolyte.^[^ ^14^, ^25^ ^]^ In the following two scans, this peak is weaker and contributes to a lower capacity than that for the first cycle. After the second scan, no obvious redox peaks can be observed but a box‐type CV shape, similar to what is generally obtained for typical (pseudo)capacitive materials.^[^ ^27^ ^]^ To analyze the reaction kinetics of MgVP/C, CV was further carried out at different scan rates from 0.05 to 5 mV s^−1^ (Figure 4b). According to Muller's work,^[^ ^28^ ^]^ the peak current obeys a power‐law relationship with the sweep rate: *i* = *av^b^*. Here *a* is a constant and *v* is the sweep rate. The *b* value can vary between 0.5 and 1.0, indicating a diffusion‐controlled process for *b* = 0.5 and a surface‐controlled process for *b* = 1.0 (generally attributed to a capacitive‐type process). The *b* value can be determined by the slope of log(*i*) versus log(*v*), as displayed in Figure 4c. In the case of MgVP/C in LIBs, *b* values are 0.81 and 0.82 at 1.0 and 1.1 V, respectively (close to 1), suggesting that it is a pseudocapacitive controlled surface process. Pseudocapacitive materials generally charge/discharge much faster than battery materials. The pseudocapacitive nature of electrode materials can guarantee fast ions diffusion kinetics, thus improving the rate performance.^[^ ^29^ ^]^ In contrast, polyanion‐type phosphate materials M_3_V_2_(PO_4_)_3_ (M = Li/Na/K) exhibit clear redox peaks and only serve as insertion‐type hosts with diffusion‐controlled kinetics as discussed in the introduction section.

In order to further clarify the reaction mechanism of MgVP/C during the discharge/charge process, in operando synchrotron diffraction was performed in the potential range of 3.0–0.01 V versus Li^+^/Li, Na^+^/Na, and K^+^/K, respectively.

**Figure 5:**
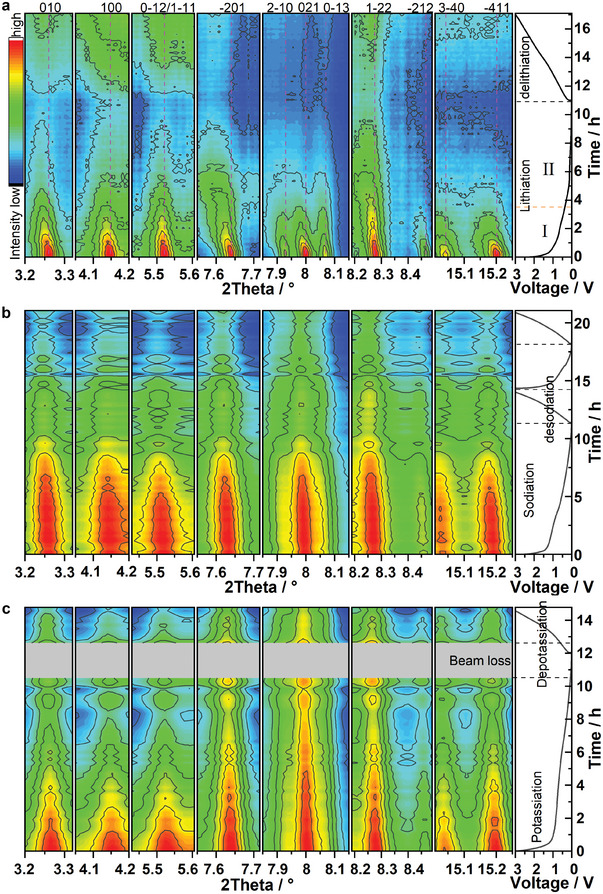
Structural evolution of MgVP/C in Li/Na/K‐ion batteries. Contour maps of in operando synchrotron diffraction patterns of MgVP/C in LIBs for the 1st cycle (ALBA, *λ* = 0.41273 Å) at 60 mA g^−1^ at a cell voltage of 0.01−3.0 V a), during the first two/one cycles for MgVP/C in SIBs b) and PIBs c), respectively, at 30 mA g^−1^ (Wavelength has been converted to *λ* = 0.41273 Å from *λ* = 0.20695 Å for convenience, which were collected at P02.1, DESY).

**Figure** **5**b,c and Figure S4 in the (Supporting Information display the in operando synchrotron diffraction of MgVP/C in both SIBs and PIBs. As shown in Figure S5a,b (Supporting Information), all reflections of MgVP/C can be indexed to triclinic MgVP with a space group of $P\bar{1}$ before cycling. During the 1st discharge (sodiation), reflections of MgVP shift to lower angles with a slight decrease in intensity, where the decrease in intensity is attributed to the formation of SEI on the electrode surface. This indicates a single‐phase solid solution reaction upon Na‐ion insertion in the structure^[^ ^30^ ^]^ whose lattice parameters *a*, *b*, and *c* exhibit a gradual increase. During the 1st charge (desodiation), these reflections shift to higher angles but do not return to their original positions, indicating the partial irreversibility of the process. This is also proven by the changes in lattice parameters (Figure S6a, Supporting Information) after desodiation in the first cycle, while it shows reversible changes of lattice parameters in the second cycle, implying better reversibility compared with the first cycle. In the case of PIBs, a similar behavior is observed when the material is cycled, but with less lattice expansion (Figure S6b, Supporting Information), implying a lower amount of K^+^ insertion than that of Na^+^ despite their similar initial capacity (sodiation/desodiation capacity of 330/79 mAh g^−1^, potassiation/depotassiation capacity of 350/77 mAh g^−1^).

In contrast, the diffraction patterns of MgVP/C in LIBs exhibit a completely different evolution as shown in the Contour maps of Figure 5a and Figure S7 in the Supporting Information, which are divided into two regions for the lithiation process. In Region I, corresponding to a lithiation capacity of 200 mAh g^−1^, all reflections of MgVP shift toward lower angles with a significant decrease in intensities. This indicates the coexistence of a two‐phase transition and solid solution, which probably can be attributed to nonequilibrium conditions after Li insertion. The material goes through an insertion reaction to form the solid solution phase of Li*_x_* Mg_3_V_4_(PO_4_)_6_ and, simultaneously, a conversion reaction to form Mg^0^, V^0^, and Li_3_PO_4_ takes place. During the solid solution reaction in Region I, the lattice parameters *a* and *b* exhibit a notable increase, while the lattice parameter *c* shows a slight increase, accompanied by the decrease of *α* and *β*, and the increase of *γ* (Figure S8, Supporting Information). This is ascribed to the Li^+^ insertion in the MgVP host, extending the lattice parameters *a*, *b*, and *c*. In Region II, no notable shifts of reflections to lower angles are observed, their intensities continuously decrease instead, suggesting a conversion reaction in this region. At the end of the lithiation process (0.01 V), only small and broad reflections of Li*_x_* Mg_3_V_4_(PO_4_)_6_ can be observed, which can be due to the high mass loading (≈7.6 mg cm^−2^) that caused an incomplete conversion reaction with a lower discharge capacity (640 mAh g^−1^) (Figure S9, Supporting Information). Unfortunately, there are no reflections of Mg^0^, V^0^, and Li_3_PO_4_ visible due to the amorphous features/low crystallinity of those phases. During the delithiation process, the reflections do not completely return to their initial positions, indicating the large irreversibility of MgVP/C during cycling. Instead, these reflections almost remain at the same intensities as those at 0.01 V with slight shifts to higher angles (Figure S9, Supporting Information). This implies the formation of Li_y_Mg_3_V_4_(PO_4_)_6_ from Mg, V, and Li_3_PO_4_ via a conversion reaction because of the low crystallinity and electrochemical pulverization. MgVP/C suffers from large structure irreversibility for three types of battery cells in the first cycle. In SIBs, it shows reversible changes of lattice parameters in the second cycle (Figure S6, Supporting Information), implying better reversibility compared with the first cycle. MgVP/C shows poor cycling stability in LIBs and a quite good cycling stability in SIBs/PIBs for the following 50 cycles. One can assume that the MgVP/C still undergoes poor reversibility in LIBs and high reversibility in SIBs/PIBs from a structural point of view. It is worth mentioning that MgVP goes through a conversion reaction and this is completely different from that of monoclinic LVP and calcium substituted LVP, which are working as insertion‐type negative electrodes upon lithiation.^[^ ^16^ ^]^ MgVP/C shows guest ion‐dependent reaction mechanisms for monovalent‐ion batteries that is clearly different from graphite and M_3_V_2_(PO_4_)_3_ (M = Li/Na/K) as mentioned above. This demonstrates that the storage mechanism of polyanion‐type phosphate in monovalent‐ion batteries depends on both the guest ion and the material crystal structure.

**Figure 6:**
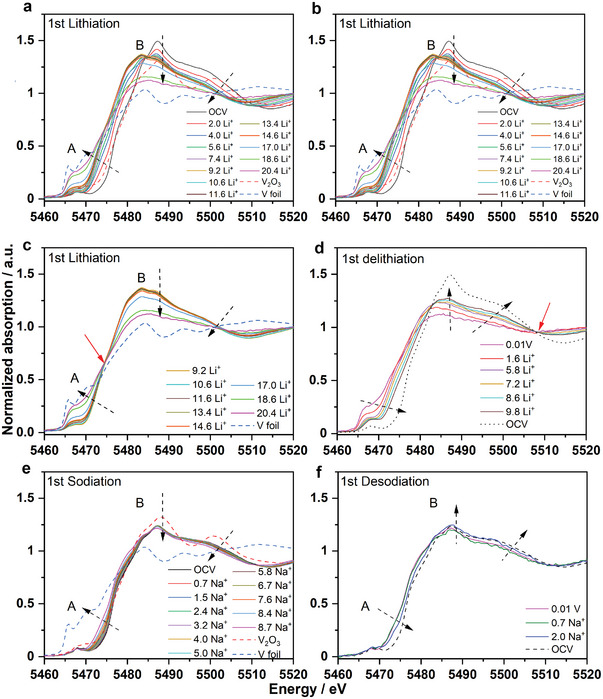
Charge compensation mechanism of MgVP/C. V K‐edge XANES spectra of MgVP/C during the 1st lithiation a–c), delithiation d) processes, the 1st sodiation e) and desodiation f) processes; isosbestic points are indicated by red arrows.

In operando XAS was conducted to probe the electronic and structural environments of V ions in MgVP/C during cycling. The normalized V K‐edge XANES spectra collected during the 1st lithiation‐delithiation processes are displayed in **Figure** **6**a–d. The edge position of the V K‐edge XANES spectrum at OCV is almost overlapping with that of the V_2_O_3_ reference, implying that the oxidation state of V in MgVP/C can be confirmed to be mainly +3, which is in good agreement with magnetic measurements below. Meanwhile, a weak pre‐edge peak (A in Figure 6a) for the V K‐edge of pristine MgVP/C is due to the transitions between the 1s and bound p‐hybridized d‐states, very similar to the spectrum of V_2_O_3_.^[^ ^31^ ^]^ In the case of the lithiation process, the graphs are grouped in two main regions (Figure 6b,c): i) from OCV (pristine state) to 0.17 V (corresponding to 9.2 Li^+^ ions lithiation) and ii) from 0.17 to 0.01 V (corresponding to the consumption of additional 11.2 Li^+^ ions). During the whole lithiation process, in total 20.4 moles of Li^+^ are consumed. During the consumption of the first 9.2 moles of Li^+^ ions, the edge position of the V K‐edge continuously shifts to lower energy. The pre‐peak (inset of Figure 6b) slightly shifts to lower energy and decreases in intensity during the consumption of the first 5.6 Li^+^ ions (0.41 V), implying the reduction of V. The edge resonance (B in Figure 6a) demonstrates notable changes in both intensity and shape, which are related to the energy absorption by core electrons.^[^ ^31^ ^]^ The edge resonance at an energy of ≈5487 eV significantly decreases its intensity along with the lithiation of 5.6 Li^+^ ions. Meanwhile, a clear peak at ≈5484 eV appears for the edge resonance, which increases its intensity with further lithiation with 9.2 Li^+^ ions. In the ranges of OCV‐4.0 Li^+^ ions (around 0.6 V) and 5.6–9.2 Li^+^ ions, two isosbestic points at 5484.4 and 5485.8 eV are observed (inset of Figure 6b), respectively, while no isosbestic point is observed in the whole range of OCV‐9.2 Li^+^ ions (around 0.17 V) during lithiation. This suggests that the intermediary product of lithiated‐Mg_3_V_4_(PO_4_)_6_, as observed from in operando synchrotron diffractions is probably attributed to nonequilibrium conditions after lithiation. Upon further lithiation from 0.17 to 0.01 V (9.2–20.4 Li^+^ ions), the edge profiles change significantly due to the deformation of V local environments (Figure 6b). At the end of lithiation, the edge position is very close to that of V foil, with the disappearance of the pre‐edge feature that is related to the formation of a large amount of V nanoparticles via conversion reaction. The slight difference between the fully lithiated electrode and V foil reference could be attributed to the isolated V nanoparticles and incomplete conversion reaction as observed via in operando synchrotron diffraction. Moreover, one isosbestic point at 5474.5 eV is observed, which further confirms the two‐phase reaction from the Li*_x_* Mg_3_V_4_(PO_4_)_6_ to Mg, V, and Li_3_PO_4_. However, even the complete conversion reaction (maximum 18 Li^+^ ions) cannot justify such a large amount of lithium, suggesting that the formation of SEI, the amorphous carbon, and the conductive additive in the electrode would consume part of the lithium in the first lithiation process. During the delithiation process, the edge position of V K‐edge XANES spectra shifts toward higher energy (Figure 6d), but cannot completely return to its original position, indicating the oxidation of V upon delithiation of 9.8 Li^+^ ions and the large irreversibility of MgVP/C during cycling.

In contrast, the normalized V K‐edge XANES spectra of MgVP/C during the 1st sodiation‐desodiation (Figure 6e,f) show a different evolution compared with that in LIBs. Only small shifts of the edge position of V K‐edge are observed during the whole sodiation process and edge resonance (B in Figure 6e) does not exhibit significant changes in its shape and intensity. During the desodiation process, the spectrum moves back to higher energy (Figure 6f), but cannot reach its original position, indicating the oxidation of V and the partial reversibility of MgVP/C. This demonstrates that MgVP/C undergoes completely different electrochemical processes in SIBs/PIBs compared with what happens in LIBs, in good agreement with in operando synchrotron diffraction.

Magnetic properties of the pristine and lithiated MgVP/C were investigated to learn about the electronic configurations of V ions and their evolution. The zero‐field cooled (ZFC) and field‐cooled (FC) curves of the pristine MgVP/C sample show predominantly Langevin type paramagnetism stemming from magnetic moments located at the V ions (Figure S10, Supporting Information). Below 5 K, it demonstrates a bifurcation of the ZFC and FC branch, implying a magnetic phase transition from the paramagnetic state to another magnetic state that exhibits some sort of irreversible magnetization behavior. However, no hysteresis effects could be observed within the experimental resolution (≈10 Oe) from the magnetic field scan at 2 K (Figure S11, Supporting Information). From Curie–Weiss fit of MgVP/C from 150 to 390 K (Figure S12, Supporting Information), Curie constant *C*, the paramagnetic effective moment *µ*_eff_, and the Weiss constant *θ* are estimated as listed in Table S2 (Supporting Information), which are in good agreement with previous work^[^ ^22^ ^]^ except that no bifurcation of the ZFC and FC curve was reported therein down to 2.5 K. In MgVP, vanadium is formally present as V^3+^ with an [Ar] 3d^2^ electronic configuration leading to a ^3^F ground and first excited ^3^P term. In ideal octahedral coordination the ^3^F ground term splits into a triplet ^3^T_1_ ground state (t^2^e^0^), a first excited triplet ^3^ *T*_2_ state (t^1^e^1^) and a nondegenerate ^3^A_2_ state (t^0^e^2^) whereas the ^3^P term only transforms into a ^3^T_1_ state. The ^3^T_1_ (^3^F) couples with the isosymmetric ^3^T_1_ (^3^P) state and an orbital momentum is partially restored. This orbital momentum reduces the spin‐only momentum (*µ*_eff,SO_ = 2.83 *µ*_B_ for *S* = 1) since spin and orbital momentum are opposed to each other for less than half filled d‐ shell and only a slightly reduced effective magnetic moment of *µ*_eff_ = 2.55(1) *µ*_B_ can be observed experimentally.

**Figure 7:**
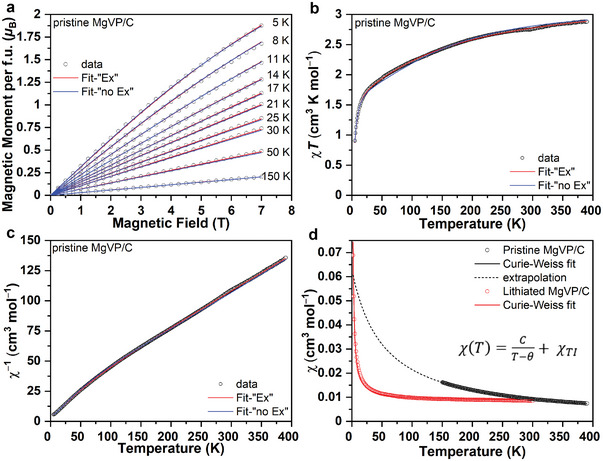
Magnetic properties of the pristine and lithiated MgVP/C. Magnetic moment versus field a), *χT* versus *T* b) and *χ* ^−1^ versus *T* c) for pristine MgVP/C together with simulated curves according to model “Ex” (red lines) and “no Ex” (blue lines); magnetic susceptibility *χ* versus *T* of pristine and lithiated MgVP/C together with Curie–Weiss fits, respectively, extrapolation d).

Beyond the extraction of Curie–Weiss parameters from the linear response, a full quantitative description of the paramagnetic state comprising all magnetic field and temperature scans for *T* > 5 K was performed using the program PHI.^[^ ^32^ ^]^ Two different magnetic models “Ex” and “no Ex” (“Ex” refers to magnetic exchange interaction) are considered with the introduction of *S* = *J* = 1 state (*J*: total orbital momentum). An isotropic effective *g*_eff_ parameter (accounting for contributions from orbital momentum) for magnetic V^3+^ site and orthorhombic crystal field splitting (CFS) parameters $D\;=\;3B_{2}^{0}\theta_{2}\;$(axial) and $E=B_{2}^{2}\theta_{2}\;$(transversal) were introduced to account for magnetic anisotropy according to the crystal field (CF) term ${\hat{H}}_{\mathrm{CF}}$ (Equation (S1), Supporting Information). The relation of the well‐known zero field splitting (ZFS) parameters *D* and *E* to the CFS parameters are given here since both (ZFS and CFS up to 2nd order) are treated by the same quantum formalism. For the magnetic model “Ex,” two of these *J* = 1 sites with constraint equal CF parameters are coupled by isotropic magnetic exchange interaction *J*_iso_ expressed by the ${\hat{H}}_{\mathrm{EX}}$ term in the Hamiltonian (Equation (S1), Supporting Information). While only a single *J* = 1 site without any exchange interaction but only CF is considered for the magnetic model “no Ex.” **Figure** **7**a–c, and Figure S13 (Supporting Information) show the fits based on magnetic model “Ex” (red lines) to the magnetization versus field and the *χT*, *χ* ^−1^, and *χ* versus temperature scans with a high degree of agreement, and the refined parameters are summarized in Table S3 (Supporting Information). However, single site model “no Ex” is capable to describe the experimental data almost equally well (blue lines in Figure 7a–c and Figure S13 in the Supporting Information and refined parameters in Table S3 in the Supporting Information), revealing that the magnetic exchange interaction does not play a crucial role but the single site magnetic anisotropy (CFS). The CFS causes the splitting of the formally degenerate *S* = *J* = 1 triplet as illustrated in Figure S14 and Equations (S2)–(S4) in the Supporting Information. The excited state *H*_n_ plays the major role in the high‐temperature region causing *χT* to increase even up to 390 K, whereas the two low lying states *G*_1m_ and *G*_2m_ determine the low temperature < 25 K properties due to the steep decrease of *χT* and the evolution of the field scans at lowest temperatures that are still far away from saturation behavior. As outlined by Görller‐Walrand et al.^[^ ^33^ ^]^ *J* = 1 states can act as a tool to probe local site symmetry by investigating their level splitting. In MgVP, the VO_6_ structural units strongly deviate from an ideal octahedron with one very short V—O bond compared to all other V—O bonds (Table S4, Supporting Information).^[^ ^22^ ^]^ This reduces the symmetry from cubic (*O*_h_) to tetragonal (*D*_4h_) expressed by the introduction of the axial asymmetry parameter *D* ($B_{2}^{0}$). Another smaller distortion within the plane perpendicular to the axis of this very short V—O bond further reduces the coordination symmetry from tetragonal to at least orthorhombic and the description needs the additional transverse *E*($B_{2}^{2}$) parameter. This orthorhombic distortion finally causes the initial ^3^T_1_ (^3^F) triplet ground state to be split into three singlet terms that create the observed magnetic anisotropy via spin–orbit coupling. These single site anisotropies are capable to explain the observed magnetic properties, i.e., the determined negative Weiss‐constant *θ* should not be ascribed to a dominant antiferromagnetic coupling^[^ ^22^ ^]^ but orthorhombic (axial and transverse) CFS.

The ZFC/FC curves of the lithiated sample still display a significantly weakened Langevin type paramagnetic behavior (Figure S15, Supporting Information) with susceptibility inversely proportional to temperature. But the susceptibility exhibits a nonlinear dependence on the applied magnetic field, i.e., the curves do not superimpose when normalized to the magnetic field. As revealed in Figure S16 (Supporting Information), a soft ferromagnetic contribution is observed, which easily saturates already at low magnetic fields and is present over the whole temperature range even at 300 K. As a consequence, the magnetic field scans do not linearly approach zero magnetization at zero field, but there is a steep increase of magnetization at low fields. This explains that the observed field dependence of the magnetic susceptibility curves, i.e., curves obtained at 1000 and 5000 Oe, for instance, do not superimpose when normalized to the magnetic field (Figure S15, Supporting Information). Therefore, the FC curve measured at 5000 Oe is utilized for further evaluation with Curie–Weiss fit (Figure 7d), and the obtained values are listed in Table S2 (Supporting Information) for the lithiated sample. The lithiated sample exhibits three different types of magnetic contributions: a) Under the assumption that the localized moments still present in the lithiated sample stem from V^2+^ with $\mu_{\mathrm{eff}}^{2+}\;$= 3.87 *µ*_B_ (compared to the pristine sample where the moments stem from V^3+^ with $\mu_{\mathrm{eff}}^{3+}$ = 2.83 *µ*_B_) according to (Equation (S5), Supporting Information), only about 2.2% of the V ions are still contributing to Langevin paramagnetism showing Curie–Weiss behavior. b) From a comparison of the maximum magnetization of the pristine sample at 2 K of about 2 *µ*_B_ that serves as a rough measure of saturation magnetization (Figure S11, Supporting Information) with the saturation magnetization of the small ferromagnetic contribution in the lithiated sample of about 0.01 *µ*_B_ (Figure S16, Supporting Information), it can be roughly estimated that only 0.01/2 = 0.5% of the V ions contribute to such a ferromagnetic phase. This ferromagnetic phase is saturated at 5000 Oe over the whole temperature range and contributes to the temperature‐independent positive susceptibility with about 1 × 10^−2^ cm^3^ mol^−1^ (Table S2, Supporting Information). This finding means that ≈2.2% of all V ions from the pristine material still contribute to the paramagnetic signal as V^2+^, ≈0.5% contribute as incorporated into a ferromagnetic phase and c) the rest and majority of V atoms (≈97%) that have reduced to metallic vanadium only contribute with 2.84 × 10^−4^ cm^3^ mol^−1^ via Pauli paramagnetism.^[^ ^34^ ^]^ The latter one is two orders of magnitude lower and quasi not visible in the magnetic data compared with the temperature independent ferromagnetic contribution.

## 3 Conclusion

MgVP/C is, for the first time, applied as a negative electrode for monovalent‐ion batteries, including LIBs, SIBs, and PIBs. MgVP/C shows a broad reduction peak at 0.7 V and a peak near 0 V for the first CV scan in LIBs. The electrode displays no obvious oxidation and reduction peaks but a box‐type CV shape and resembles a capacitive‐like behavior so that MgVP/C is classified as new pseudocapacitive material. MgVP/C delivers a high initial lithiation/delithiation capacity of 961/607 mAh g^−1^ and a reversible capacity of over 547 mAh g^−1^ in the following five cycles at 50 mA g^−1^, while the material shows much lower initial sodiation/desodiation and potassiation/depotassiation capacities of 236/63 and 301/64 mAh g^−1^. This suggests that MgVP/C is a promising negative electrode material in LIBs for practical application due to its high initial reversible capacity. However, further effort should be made to improve the poor electrochemical cycling stability and low initial Coulombic efficiency using electrolyte engineering, nanoengineering and surface coating. The combination of in operando and ex situ studies demonstrates that MgVP/C undergoes an indirect conversion reaction to form Mg^0^, V^0^, and Li_3_PO_4_ in LIBs, while the material only experiences a solid solution in both SIBs and PIBs with the reduction of V^3+^ to V^2+^. This result reveals that, due to the size differences of alkali ions, a different electrochemical mechanism occurs among Li^+^ and Na^+^/K^+^ storage, leading to the large difference in capacity. Moreover, magnetic measurements demonstrate that in the pristine MgVP, the V ions are present as V^3+^ and that strong magnetic anisotropies induced by highly distorted VO_6_ octahedra are responsible for the observed paramagnetic properties. After lithiation, most of the V^3+^ ions (≈97%) are reduced to metallic vanadium, ≈0.5% contribute to a ferromagnetic signal and ≈2.2% still contribute to a paramagnetic signal. With this work, we discovered a new material that can be used for high energy/high power devices (as this features capacitive‐like behavior) and most importantly, this work also provides an advanced understanding of polyanion‐type phosphate negative electrode material (whose storage mechanism depends on the guest ion) for monovalent‐ion batteries.

## 4 Experimental Section

### Synthesis of Mg_3_V_4_(PO_4_)_6_/C

Mg_3_V_4_(PO_4_)_6_/C was synthesized by ball‐milling (Planetary Micro Mill, PULVERISETTE 7, premium line, FRITSCH) and carbon‐thermal reduction method. Typically, starting materials Mg(CH_3_COO)_2_, NH_4_VO_3_ (9.80 mmol), NH_4_H_2_PO_4_ (14.70 mmol), and 20 mL of ethanol were mixed with a molar ratio of (Mg:V:P = 3:4:6), together with polyacrylic acid (PAA, 0.29 mmol) and d‐(+)‐ glucose (1.12 mmol). Meanwhile, PAA and d‐(+)‐glucose are used as carbon sources and reducing agents. The above mixture was ball‐milled for 8 h at 350 rpm and the procedure was set to pause for 7 min every 5 min to avoid the heating of the solution, where 180 zirconium dioxide (ZrO_2_) spheres with a diameter of 5 mm are used. After ball‐milling, the wet mixture was dried under N_2_ atmosphere at 60 °C for 12 h. The remaining powder was ground with mortar and pestle for 1 h, followed by annealing in two steps under Ar:H_2_ (95:5) atmosphere flow. The first step was carried out at 350 °C for 5 h (heating rate of 5 °C min^−1^); the second step was performed at 800 °C for 8 h with a heating rate of 3 °C min^−1^. The final black powder was ground with mortar for 1 h.

### Structural and Morphological Characterization

Crystal Structure of Mg_3_V_4_(PO_4_)_6_/C was investigated using synchrotron radiation at the beamline P02.1 at DESY in Hamburg (*λ* = 0.20723 Å, 60 keV).^[^ ^35^ ^]^ The powder sample was measured under a glass capillary geometry (0.5 mm in diameter). The diffraction data were analyzed by the Rietveld method using the Fullprof software package.^[^ ^36^ ^]^ The morphology of materials was studied with a Zeiss Supra 55 Scanning Electron Microscope (SEM). The carbon content in Mg_3_V_4_(PO_4_)_6_/C was studied by thermogravimetric analysis (TGA) on STA 449C (Netzsch GmbH) under O_2_ flow. *Specific surface area and porosity*: The Brunauer–Emmett–Teller method^[^ ^37^ ^]^ was used to estimate the specific surface areas (SSA_BET_) of Mg_3_V_4_(PO_4_)_6_/C material, which was heated at 573 K for 48 h and degassed at ≈368 K for 96 h before measuring the molecular adsorption on the particles. During the degassing process, about 13% mass loss was recorded. Argon gas at 87.3 K was used as the adsorbate. Ar provides better adsorption for the estimation of SSA because of its monatomicity and nonlocalization of the adsorbent during adsorption.^[^ ^38^ ^]^ The gas sorption was carried out with an Autosorb 1‐MP Instrument (Quantachrome GmbH, Germany). The pore size volume distribution, but also the specific surface area, denoted as SSA_DFT_, were calculated with models based on DFT/Monte Carlo methods assuming a mixture of spherical and cylindrical pores on a carbon‐based substrate.^[^ ^39^ ^]^

Transmission electron microscopy (TEM) imaging was acquired by FEI Titan under 300 kV.

Direct current magnetic moment was measured using a DynaCool Physical Property Measurement System (PPMS) from Quantum Design equipped with a vibrating sample magnetometer (VSM). Zero‐field cooled (ZFC) and field‐cooled (FC) magnetic moment versus temperature scans were measured at a field of 1000 Oe (and 5000 Oe for the lithiated sample) with a temperature resolution of 1 K in settle mode from 2 to 50 K and in sweep mode from 50 to 300 K at a rate of 2 K min^−1^. An additional FC measurement up to 390 K was measured for the pristine Mg_3_V_4_(PO_4_)_6_ sample. Full loop (without virgin curve) magnetic moment versus field measurements were measured at 2 K with maximum field of ±7 Tesla with high resolution for investigation of potential hysteretic effects. Magnetic field scans up to 7 Tesla and back to zero field with a resolution of 2500 Oe were measured at 5, 8, 11, 14, 17, 20, 25, 30, 50, and 150 K (and 300 K for the lithiated sample). Pristine sample: 11.7924 mg of Mg_3_V_4_(PO_4_)_6_ powder and 0.6076 mg (4.9 wt%) carbon; Lithiated sample: A mixture of 70 wt% pristine sample and 20 wt% carbon and 10% PVDF were lithiated so that the Mg_3_V_4_(PO_4_)_6_ effectively took up 16 Li. Accordingly, 10.2 mg of that lithiated sample contain 7.064 mg “Mg_3_V_4_(PO_4_)_6_ + 16 Li” and 3.136 mg carbon and PVDF. Carbon reference: 3.12 mg Timcal‐C65 carbon as a reference. Samples were filled into polypropylene sample capsules (Quantum Design QDS‐P125E). Due to the strong paramagnetic signal stemming from the V ions all diamagnetic contributions (from carbon, binder, filled electron shells of atoms and sample holder) can be neglected.

### Electrochemical Characterization

The working electrode was prepared by mixing active material Mg_3_V_4_(PO_4_)_6_/C, C65, polyvinylidene fluoride (PVDF) binder in a ratio of 7:2:1 dissolved in N‐methyl‐2‐pyrrolidone (NMP). The obtained slurry was coated on an Al‐foil of 15 µm (Cu foil for LIBs) and dried at room temperature for 12 h, and then dried at 80 °C for 12 h. The coated electrodes were cut into circular pieces with a diameter of 12 mm (1.131 cm^2^, ≈1.5 mg cm^−2^, thickness of ≈30 µm) and dried in a vacuum oven (Büchi Labortechnik GmbH) at 120 °C for about 12 h to remove the moisture. C65 electrode was prepared using C65 (Timcal) and polytetrafluoroethylene (PTFE, 60 wt% solution in water from Sigma‐Aldrich) in a ratio of 8:2 with solvent isopropanol in a DAC150.1 FVZ model from SpeedMixer with 800 rpm for 10 min. The paste mixture was kneaded manually on a glass plate and was finally rolled to an electrode with uniform thickness. The electrodes (1.131 cm^2^, ≈5.5 mg cm^−2^) were cut into circular pieces with a diameter of 12 mm and were finally dried at 110 °C overnight under vacuum. Then, the dried electrodes were hermetically transferred to an Ar‐filled glovebox (MBRAUN GmbH). The electrochemical performance of the negative materials was studied in two‐electrode Swagelok cells, where metallic lithium, sodium or potassium was used as a counter electrode with a Whatman glass fiber separator. The electrolytes (150 µL) were 1 m LiPF_6_ in EC/DMC (1:1 wt%, LP30, BASF), 1 m NaClO_4_ in a mixture of ethylene carbonate (EC):propylene carbonate (PC) (1:1 by volume) and 5% fluoroethylene carbonate (FEC), and 1 m potassium bis(fluorosulfonyl)imide (KFSI) in a mixture of EC:diethyl carbonate (DEC) (1:1 by volume) for LIBs, SIBs and PIBs, respectively. Cyclic voltammetry (CV) and galvanostatic cycling with potential limitation (GCPL) were performed in the potential window of 3 and 0.01 V. The CV measurement was performed at different scan rates from 0.05 to 2 mV s^−1^. All electrochemical measurements (CV and GCPL) were done on a VMP3 multichannel potentiostat (Biologic Science Instrument) with an EC‐lab software for instrument operation and data evaluation.

### In Operando Synchrotron Diffraction and In Operando X‐Ray Absorption Spectroscopy (XAS)

In operando synchrotron diffraction was performed at Material Science and Powder Diffraction beamline (MSPD) at ALBA, Spain^[^ ^40^ ^]^ and at PETRA‐III beamline P02.1 at DESY in Hamburg, Germany.^[^ ^35^ ^]^ The electrochemical cells consist of 2025‐type coin cells with glass windows of 5 mm diameter for beam entrance. The negative electrode (active materials, 2 mg, and ≈7 mg cm^−2^) was prepared by pressing the electrode mixture on Cu mesh within a 5 mm hole in the center. In operando synchrotron diffraction was conducted at ALBA with radiation wavelength *λ* = 0.41273 Å (30 keV) and position sensitive detector MYTHEN, where data were collected with an effective exposure time of 60 s in steps of 0.006° over the angular range of 1.8°–42° in 2theta during the first cycles at 60 mA g^−1^ for LIBs. In operando synchrotron diffraction was conducted at DESY/PETRA‐III at beamline P02.1 with radiation wavelength *λ* = 0.20695 Å (60 keV), where data were collected with an effective exposure time of 40 s during the first cycles at 30 mA g^−1^ for SIBs and PIBs. DAWN software is used to process the 2D powder diffraction.^[^ ^41^ ^]^ The diffraction data analysis was carried out by the Rietveld method using the Fullprof software package.^[^ ^36^ ^]^ In operando X‐ray absorption spectroscopy (XAS) measurements were carried out at PETRA‐III beamline P64 and P65 at DESY in Hamburg during the first cycle at 80 mA g^−1^ for LIBs and at 30 mA g^−1^ for SIBs between 3 and 0.01 V. XAS spectra were recorded in quick‐XAS (5 min/spectrum) mode in fluorescence geometry using a PIPS diode. The V K‐edge of Mg_3_V_4_(PO_4_)_6_/C was measured during the electrochemical cycling and the energy was calibrated using the absorption edge of V foil, as it is commonly employed in XAS experiments. All data were collected at room temperature with a Si (111) double crystal monochromator and all XAS spectra were processed using DEMETER software package.^[^ ^42^ ^]^

## Conflict of Interest

The authors declare no conflict of interest.

## Author Contributions

Q.F. conceived the idea, designed the experiments, and discussed it with B.S., Z.D., A.S., P.W., A.M., M.E., E.W., W.H., M.K., H.E., and S.D.; Q.F. performed material synthesis, characterizations, electrochemical measurements, and analyzed the data. B.S. carried out magnetic measurements and analyzed the data. Z.D. and P. W. performed TEM and BET, respectively, and discussed the data with Q.F. Q.F., A.S., A.M., M.E., E.W., and W.H. carried out in operando synchrotron diffraction/XAS and analyzed the data with M.K., H.E., and S.D. Q.F. wrote the preliminary draft with input from B.S. All authors contributed to interpreting the findings, reviewing, and commenting on the manuscript.

## Supporting information

Supporting Information

## Data Availability

The data that support the findings of this study are available from the corresponding author upon reasonable request.
