# Supplementary material for: Guest Ion‐Dependent Reaction Mechanisms of New Pseudocapacitive Mg3V4(PO4)6/Carbon Composite as Negative Electrode for Monovalent‐Ion Batteries
Source: Adv Sci (Weinh). 2023 Feb 15;10(11):2207283. doi: 10.1002/advs.202207283 (PMC10104641; doi:10.1002/advs.202207283)
Supplement: Supplementary file 1 — Supporting Information [file ADVS-10-2207283-s001.pdf]

ADVANCED SCIENCE
Open Access

# Supporting Information

for *Adv. Sci.*, DOI 10.1002/advs.202207283

## Guest Ion-Dependent Reaction Mechanisms of New Pseudocapacitive $Mg_3V_4(PO_4)_6$/Carbon Composite as Negative Electrode for Monovalent-Ion Batteries

*Qiang Fu*, Björn Schwarz, Ziming Ding, Angelina Sarapulova, Peter G. Weidler, Alexander Missyul, Martin Etter, Edmund Welter, Weibo Hua, Michael Knapp, Sonia Dsoke and Helmut Ehrenberg*

# Guest Ion-Dependent Reaction Mechanisms of New Pseudocapacitive $Mg_3V_4(PO_4)_6$/Carbon Composite as Negative Electrode for Monovalent-Ion Batteries

Qiang Fu[a,*], Björn Schwarz[a], Ziming Ding[b,g], Angelina Sarapulova[a], Peter G. Weidler[c], Alexander Missyul[d], Martin Etter[e], Edmund Welter[e], Weibo Hua[a,f], Michael Knapp[a], Sonia Dsoke[a], Helmut Ehrenberg[a]

[a]Institute for Applied Materials (IAM), Karlsruhe Institute of Technology (KIT), Hermann-von-Helmholtz-Platz 1, D-76344 Eggenstein-Leopoldshafen, Germany

[b]Institute of Nanotechnology (INT), Karlsruhe Institute of Technology (KIT), Hermannvon-Helmholtz-Platz 1, D-76344 Eggenstein-Leopoldshafen, Germany

[c]Institute of Functional Interfaces (IFG), Chemistry of Oxidic and Organic Interfaces (COOI), Karlsruhe Institute of Technology (KIT), Hermann-von-Helmholtz-Platz 1, D-76344 Eggenstein-Leopoldshafen, Germany

[d]CELLS-ALBA Synchrotron, E-08290 Cerdanyola del Valles, Barcelona, Spain

[e]Deutsches Elektronen-Synchrotron (DESY), Notkestr. 85, Hamburg 22607, Germany

[f]School of Chemical Engineering and Technology, Xi'an Jiaotong University, Xi'an, Shaanxi 710049, People's Republic of China

[g]Technische Universität Darmstadt, 64289 Darmstadt, Germany

Corresponding author: qiang.fu@kit.edu (Q. Fu)

Tel: 49-721 608-41445, Fax: 49-721 608-28521.

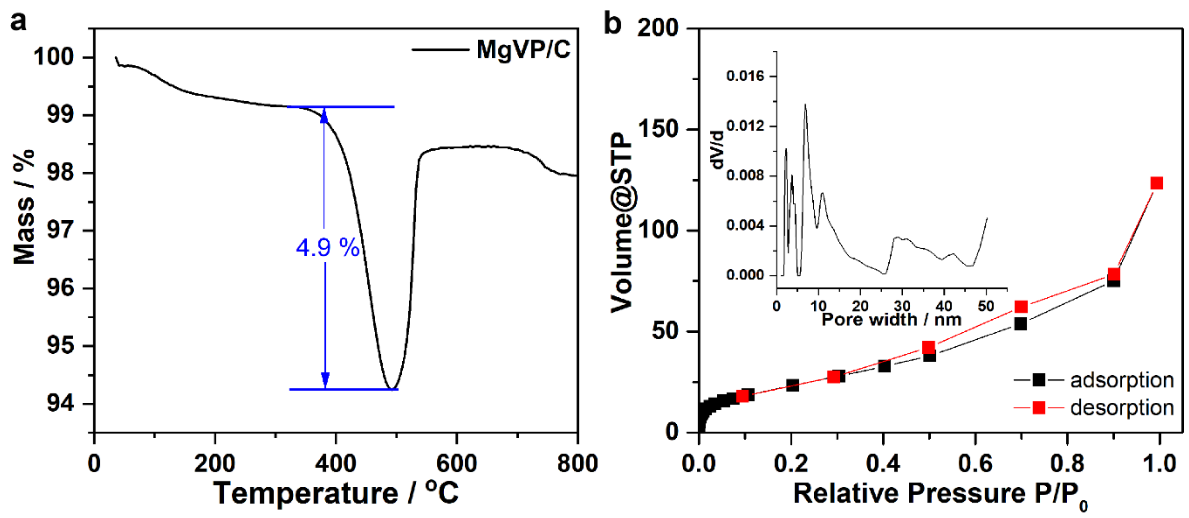


**Figure S1** TGA curve under $O_2$ flow. (a) and argon adsorption-desorption isotherms (inset pore size distribution) (b) of pristine MgVP/C.

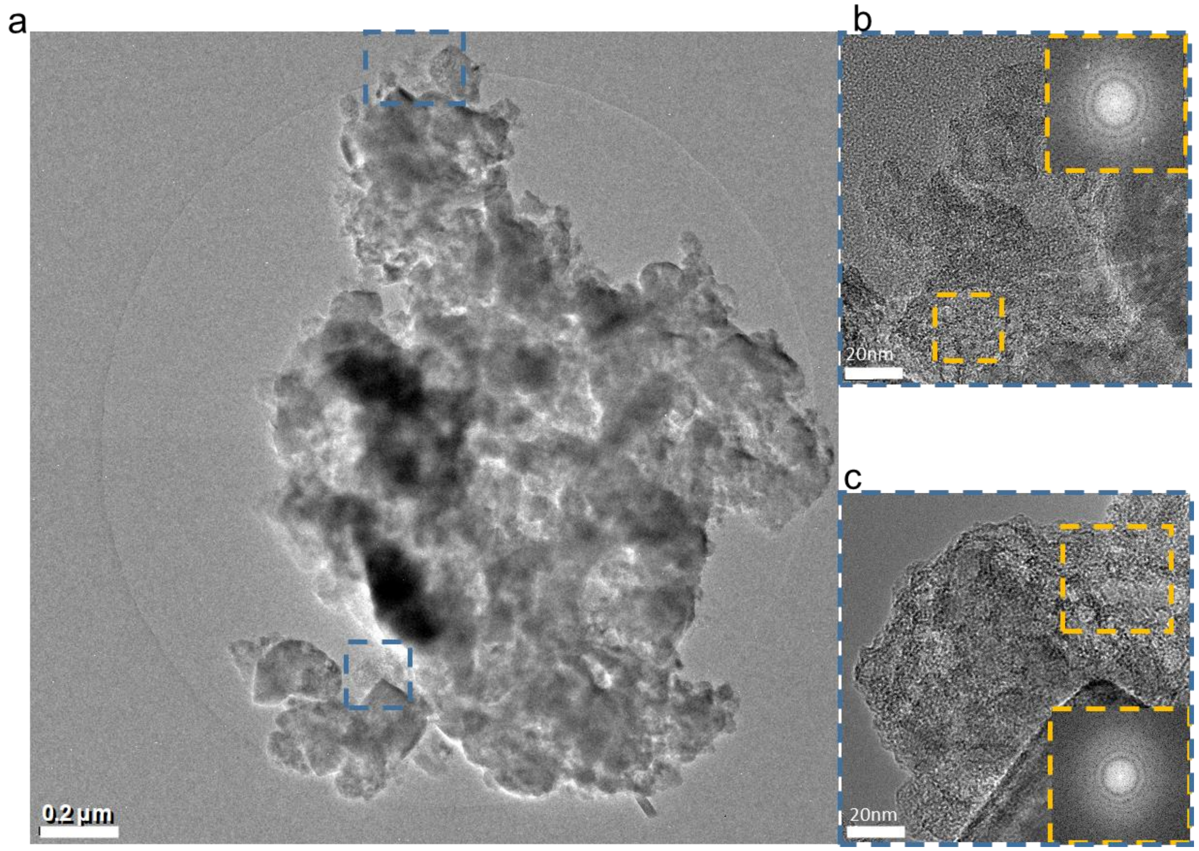


**Figure S2** TEM images of pristine MgVP/C, (b) & (c) HRTEM images of the blue inset dashed rectangle in (a).

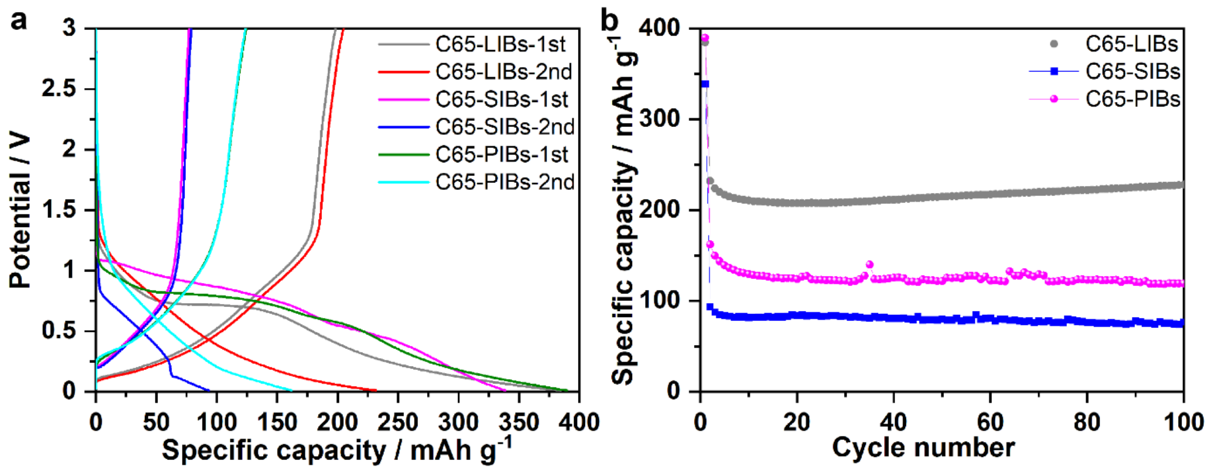


**Figure S3 Li, Na and K insertion performance in C65 conductive additive.** The discharge-charge curves (a) and cycling performance (b) of C65 at 50 mA $g^{-1}$ at 25 °C for LIBs, SIBs, and PIBs, respectively.

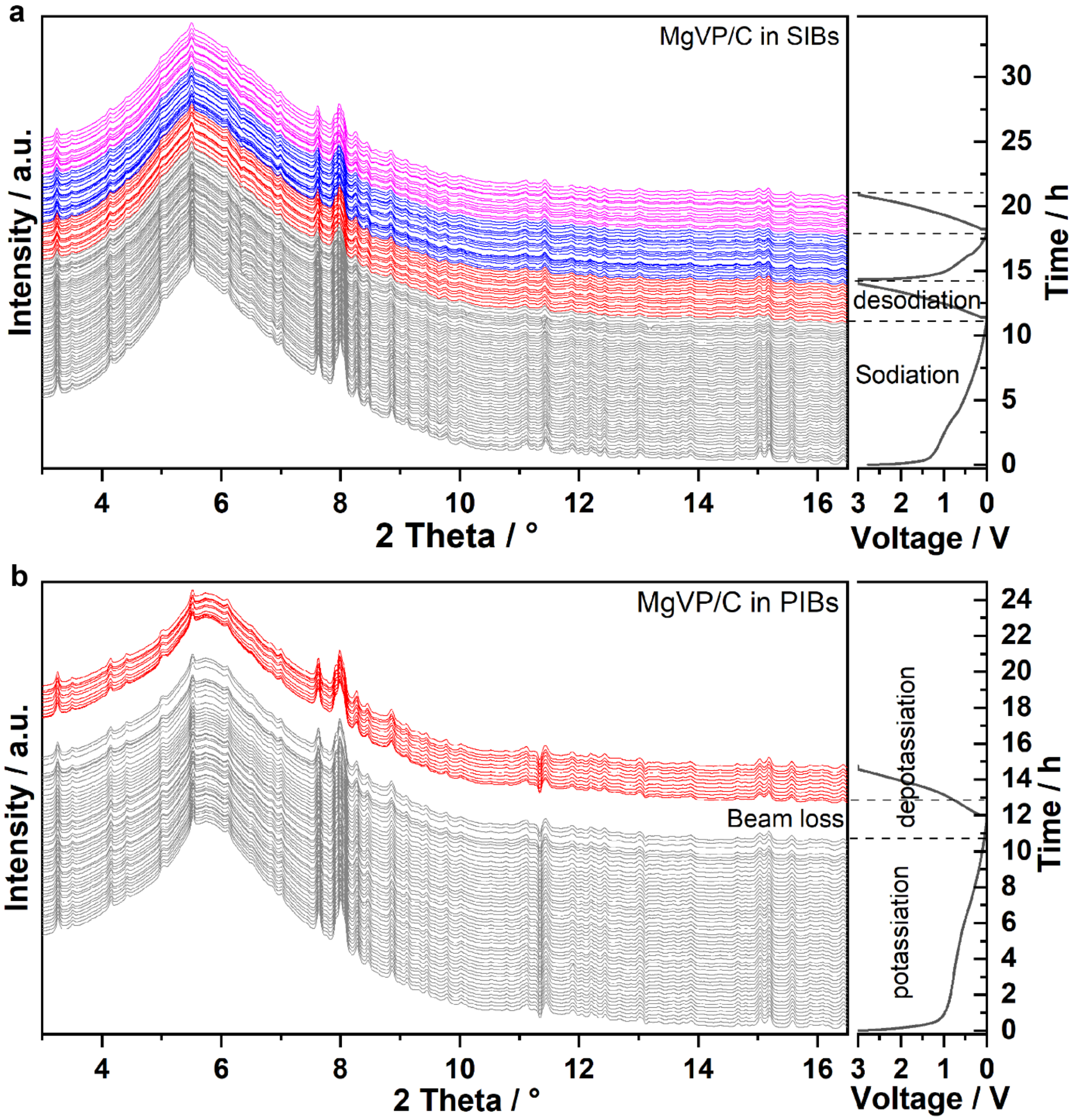


**Figure S4** *In operando* synchrotron diffraction patterns of MgVP/C during the first two/one cycles for MgVP/C in SIBs (a) and PIBs (b), respectively, at 30 mA $g^{-1}$ (Wavelength has been converted to λ= 0.41273 Å from λ= 0.20695 Å for convenience, which were collected at P02.1, DESY).

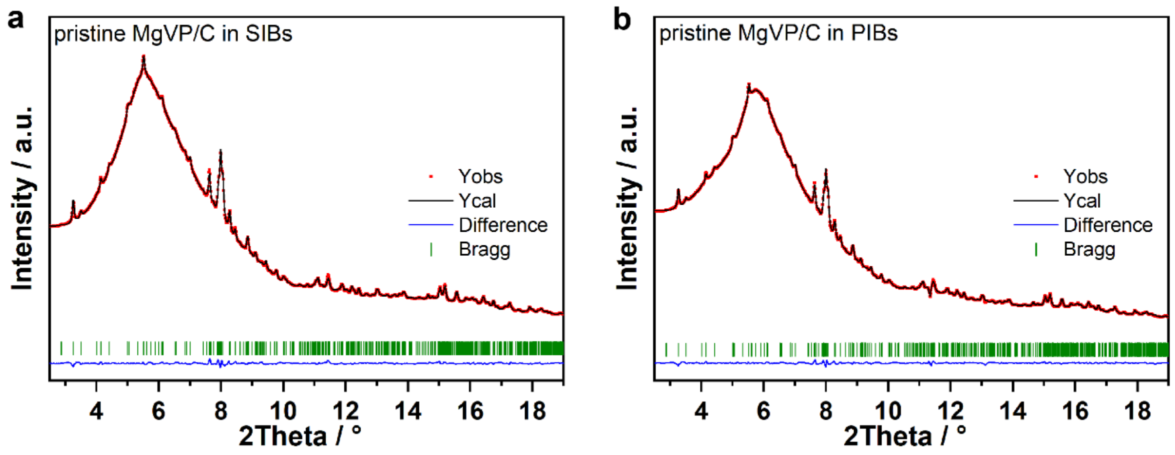


**Figure S5** Rietveld refinement from synchrotron diffraction data of pristine MgVP/C in both SIBs (a) and PIBs (b) (λ= 0.41273 Å converted from λ= 0.20695 Å).

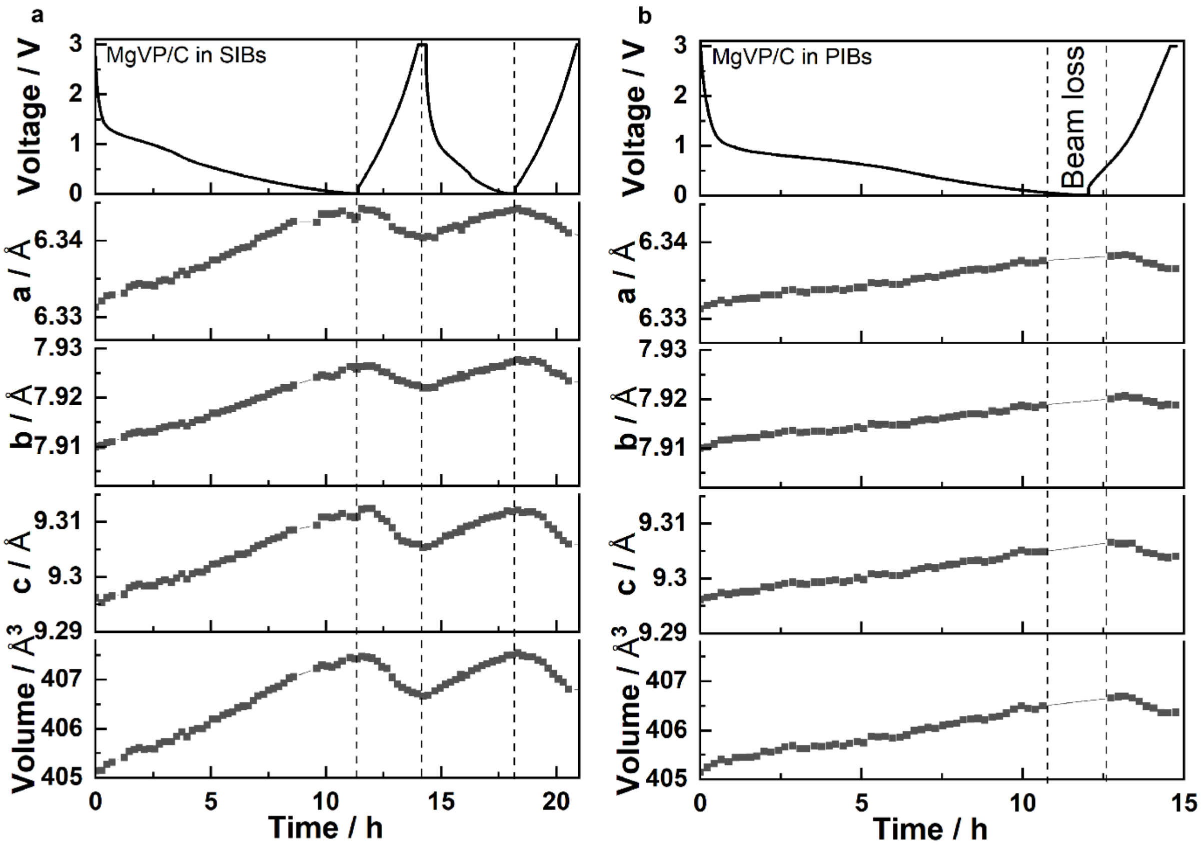


**Figure S6** Structural parameters from diffraction patterns with Rietveld refinement during the first two/one cycles for MgVP/C in SIBs (a) and PIBs (b), respectively.

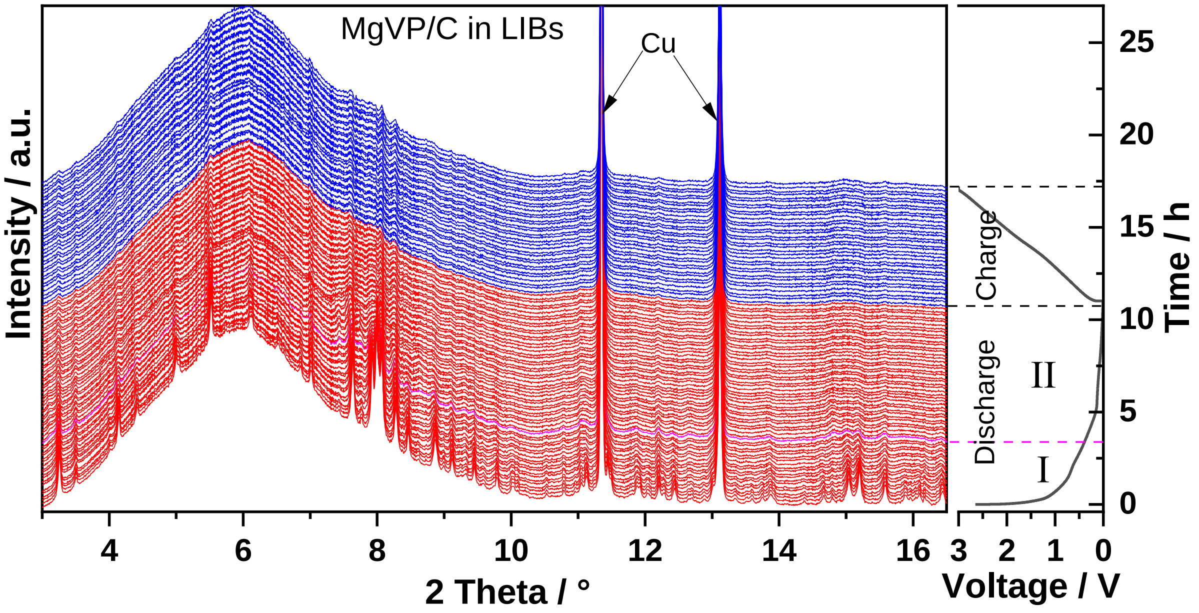


**Figure S7** *In operando* synchrotron diffraction patterns of MgVP/C in LIBs for the 1st cycle (λ= 0.41273 Å).

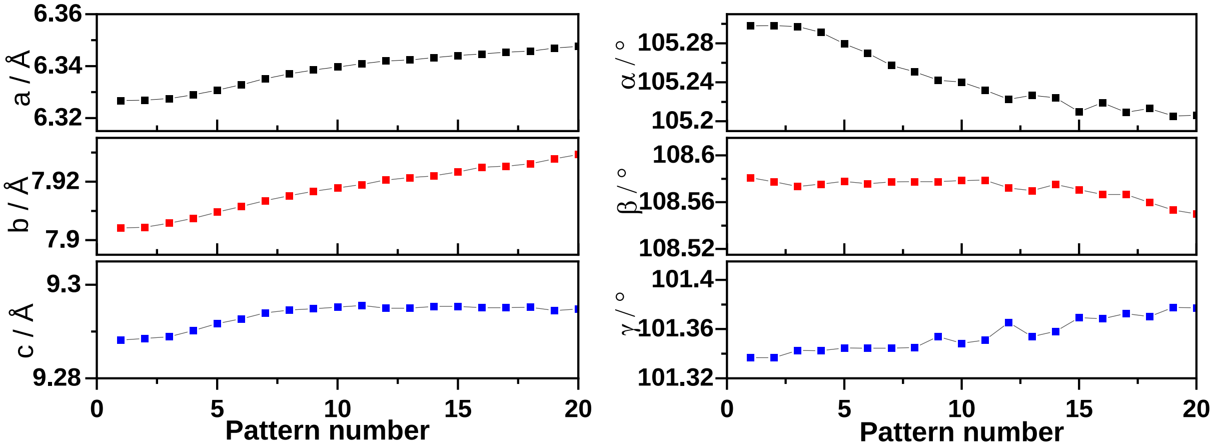


**Figure S8** Structural parameters from diffraction patterns with Rietveld refinement during the first cycle for MgVP/C in LIBs.

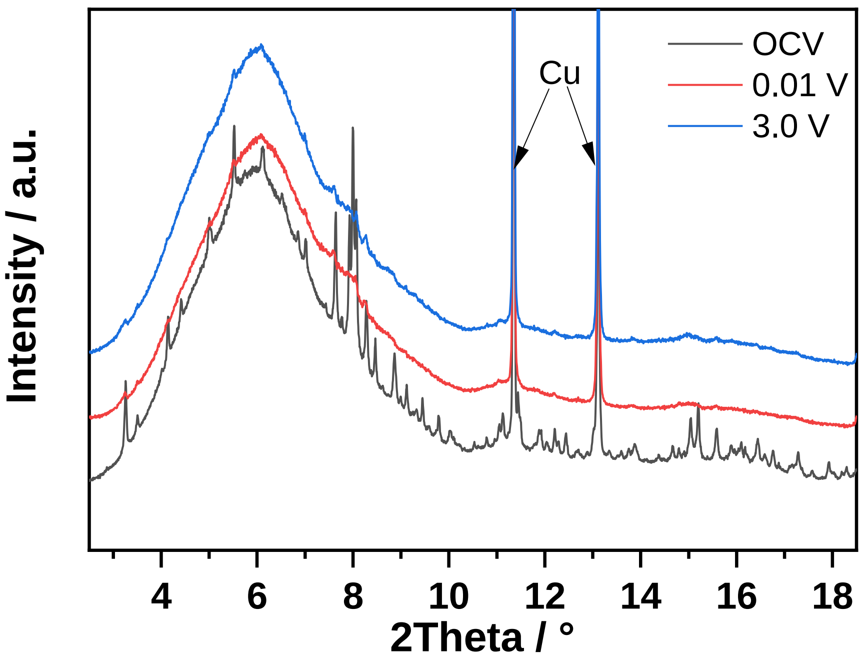


**Figure S9** Comparison of MgVP/C in LIBs at OCV, 0.01 V, and 3.0 V.

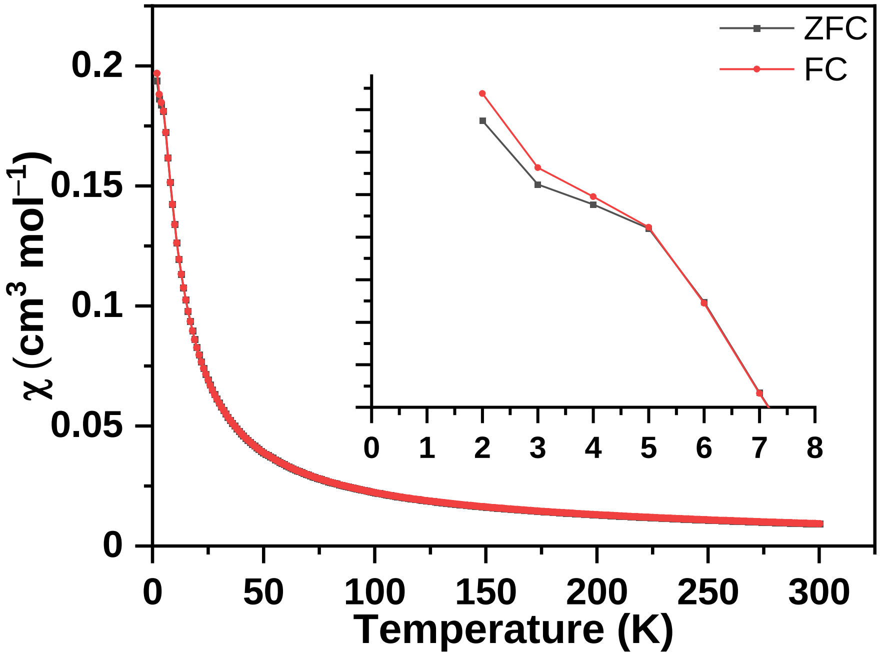


**Figure S10** ZFC and FC susceptibility $\chi$ vs. $T$ of pristine MgVP/C obtained at 1000 Oe.

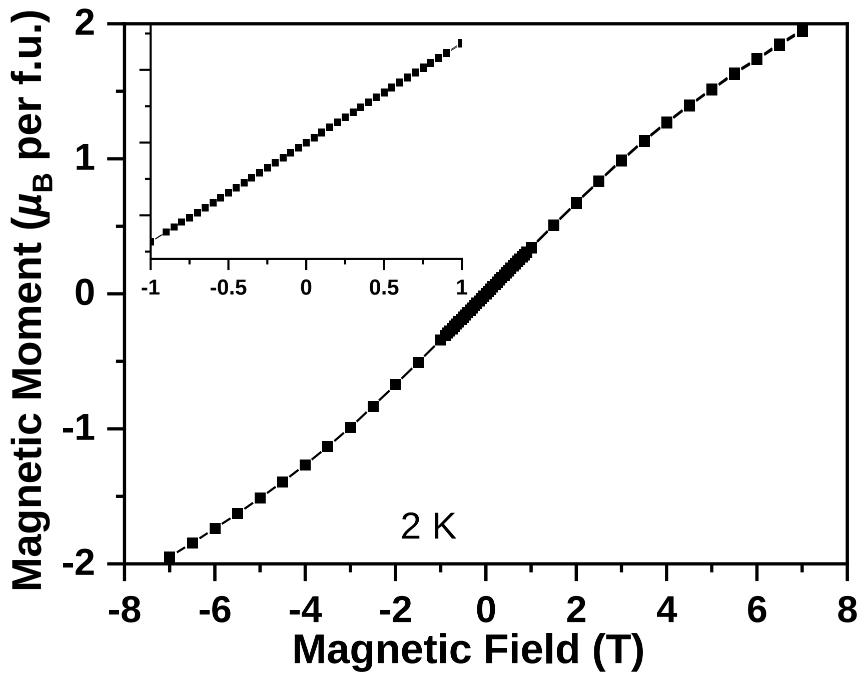


**Figure S11** Magnetic moment vs. magnetic field of pristine MgVP/C obtained at 2 K.

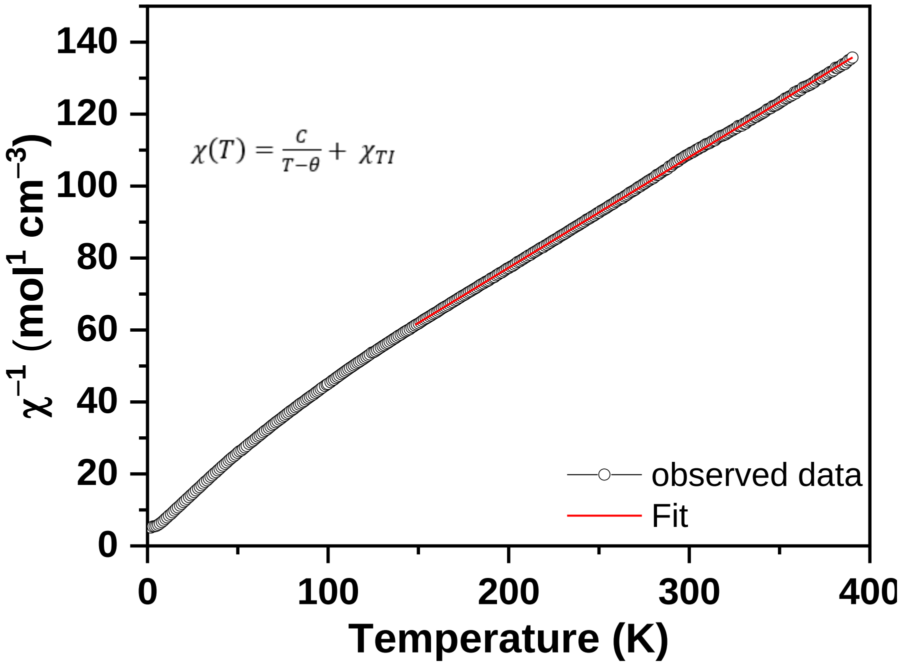


**Figure S12** Inverse susceptibility $\chi^{-1}$ vs. $T$ (open circles) for pristine MgVP/C together with Curie-Weiss fit from 150 to 390 K (solid line), where $\chi$, $C$, $T$, $\theta$ and $\chi_{TI}$ refer to molar susceptibility, molar Curie constant, Temperature, Weiss constant, and temperature independent susceptibility, respectively.

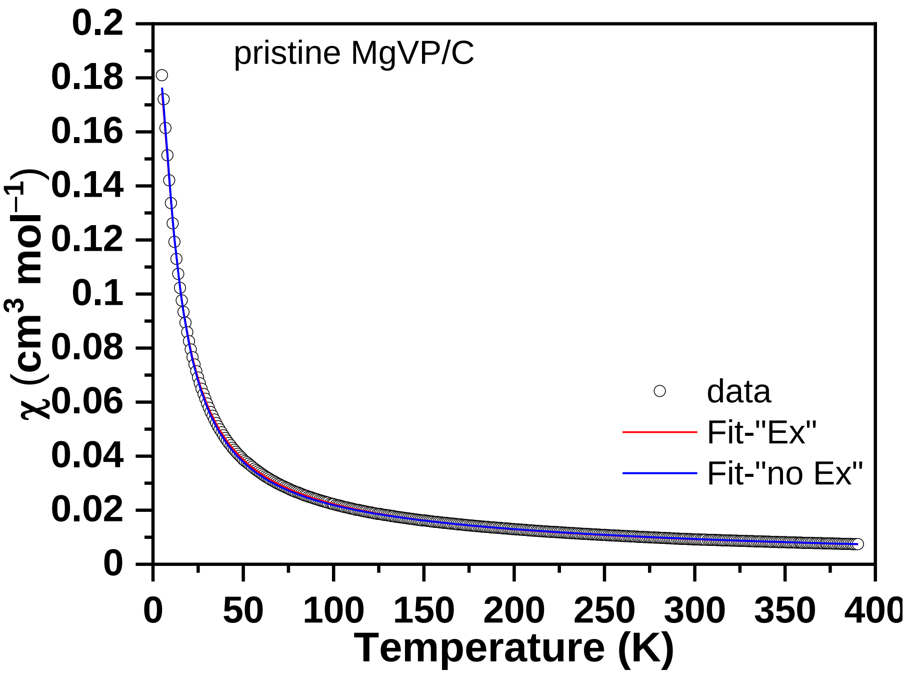


**Figure S13** susceptibility $\chi$ vs. $T$ for pristine MgVP/C together with simulated curves according to model 'Ex' (red lines) and 'no Ex' (blue lines).

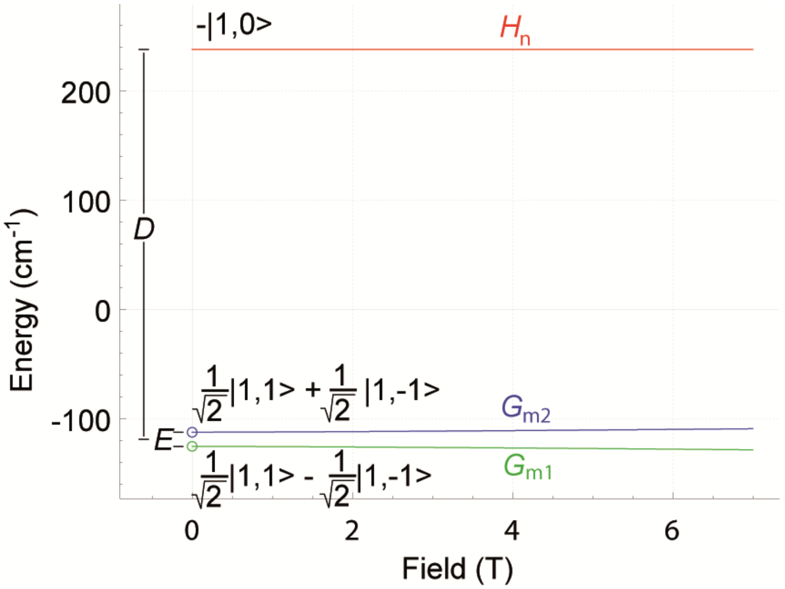


**Figure S14** Spin quantum states' energy levels as a function of magnetic field (Zeeman) according to model 'no Ex' as obtained from a fit to experimental dc magnetization vs. field and vs. temperature data. The positive axial parameter $D$ firstly causes the $m_l = 0$ state (equation S4) to be excited by the energy $D$ above the $m_l = \pm 1$ states. The transverse parameter $E$ then causes a quantum mechanical mixing of the $m_l = \pm 1$ states into $G_{1m}$ (equation S2) and $G_{2m}$ (equation S3). These two states are separated by energy $E$ that is much smaller compared to $D$.

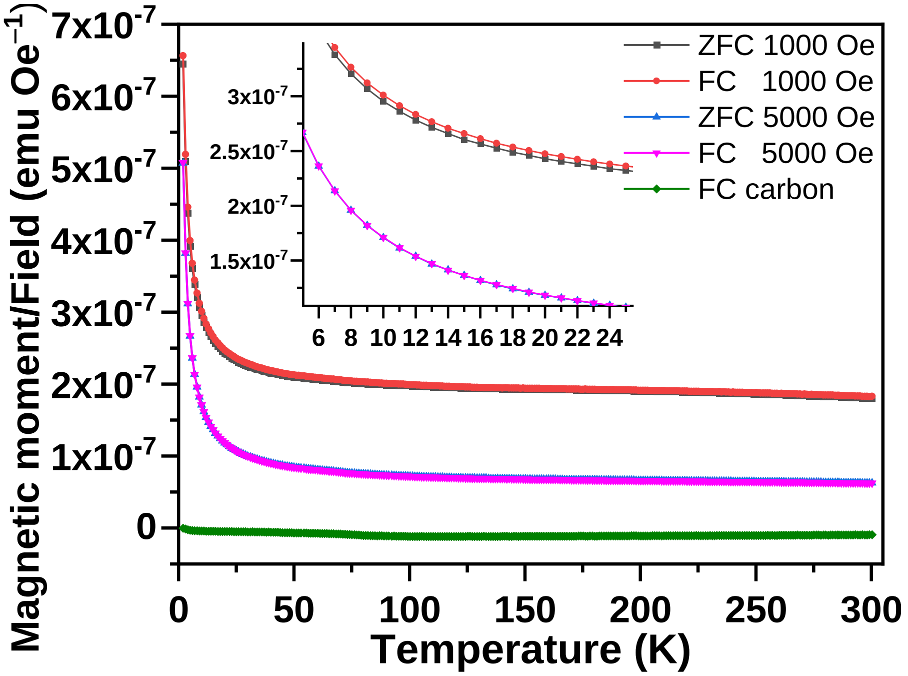


**Figure S15** ZFC/FC magnetization vs. temperature of lithiated MgVP/C obtained at 1000 and 5000 Oe, respectively, and FC curve for carbon obtained at 1000 Oe (inset).

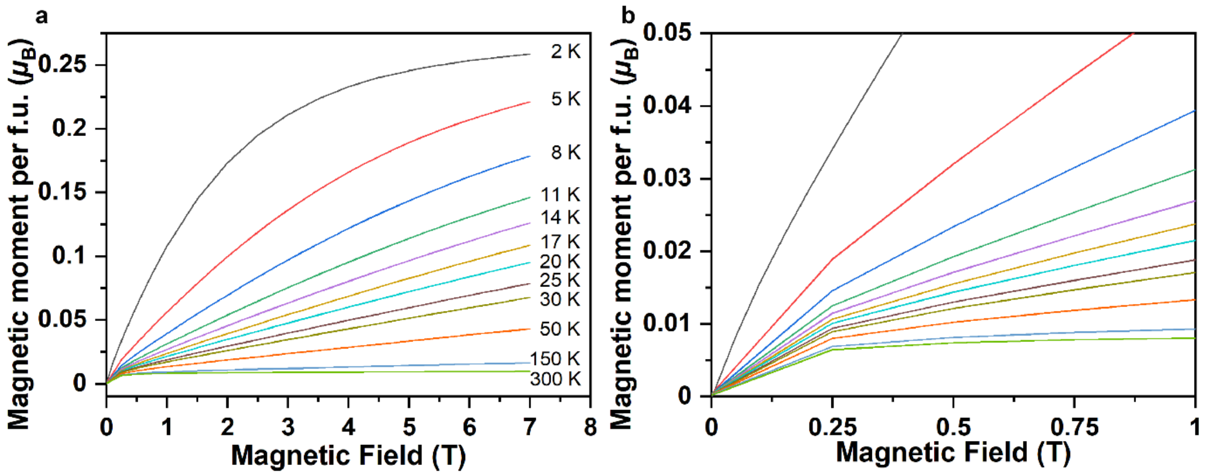


**Figure S16** Magnetization vs. filed (a) and enlarged selection (b) of lithiated MgVP/C obtained for various temperatures.

**Table S1** Crystallographic data for pristine $Mg_3V_4(PO_4)_6$

| Space group: $P\bar{1}$ (No.2)<br>$a$= 6.325(1) Å, $b$= 7.903(1) Å, $c$=9.286(1) Å<br>$\alpha$= 105.287(3)°, $\beta$=108.567(3)°, $\gamma$= 101.339(3)° | | | | | Chi=0.18<br>R-factor=2.94<br>$R_f$-factor=1.99 |
|---|---|---|---|---|---|
| Atom | Wyckoff | x | y | z | SOF |
| Mg2 | 2i | 0.28330 | 0.81370 | 0.28950 | 1 |
| V1 | 1a | 0 | 0 | 0 | 0.2620 |
| Mg1 | 1a | 0 | 0 | 0 | 0.7380 |
| V2 | 2i | 0.38850 | 0.46280 | 0.11560 | 0.9340 |
| Mg3 | 2i | 0.38850 | 0.46280 | 0.11560 | 0.0660 |
| V3 | 2i | 0.95250 | 0.28480 | 0.47840 | 0.9340 |
| Mg4 | 2i | 0.95250 | 0.28480 | 0.47840 | 0.0660 |
| P1 | 2i | 0.22440 | 0.14220 | 0.76940 | 1 |
| P2 | 2i | 0.08790 | 0.58930 | 0.83440 | 1 |
| P3 | 2i | 0.60870 | 0.76940 | 0.63290 | 1 |
| O1 | 2i | 0.22510 | 0.19830 | 0.94480 | 1 |
| O2 | 2i | 0.54580 | 0.62090 | 0.71530 | 1 |
| O3 | 2i | 0.01330 | 0.18560 | 0.65900 | 1 |
| O4 | 2i | 0.10490 | 0.78780 | 0.91840 | 1 |
| O5 | 2i | 0.73380 | 0.96280 | 0.76890 | 1 |
| O6 | 2i | 0.37330 | 0.77340 | 0.50900 | 1 |
| O7 | 2i | 0.05720 | 0.54320 | 0.64970 | 1 |
| O8 | 2i | 0.45660 | 0.25960 | 0.77070 | 1 |
| O9 | 2i | 0.87340 | 0.45790 | 0.83680 | 1 |
| O10 | 2i | 0.76060 | 0.72220 | 0.53730 | 1 |
| O11 | 2i | 0.20960 | 0.94010 | 0.70630 | 1 |
| O12 | 2i | 0.30880 | 0.53980 | 0.91850 | 1 |

**Table S2** Parameters obtained from Curie-Weiss fit to pristine and discharged MgVP/C.

| | Pristine MgVP/C | Discharged MgVP/C |
|---|---|---|
| Molar Curie constant $C_{mol}$ ($cm^3$ K $mol^{-1}$) | 3.257(3) | 0.1316(7) |
| paramagnetic effective moment $\mu_{eff}$ ($\mu_B$) | 5.10(1) (2.55(1) per V) | - |
| Weiss constant $\theta$ (K) | –51.8(3) K | 0 (not refined) |
| Temperature independent sus. $\chi_{TI}$ ($cm^3$ $mol^{-1}$) | 0 (not refined) | $8.48(3)\cdot10^{-3}$ ($\sim 1\cdot10^{-2}$) |

**Table S3** Results of refinement to magnetic dc data according to model 'Ex' and 'no Ex'.

| | Parameters **model 'Ex'** | | | Parameters **model 'no Ex'** | | |
|---|---|---|---|---|---|---|
| | | value | remark | | value | remark |
| Total orbital quantum nr. $J = S$ | | 1 | fixed | | 1 | fixed |
| Isotropic exchange $J_{iso}$ | #1 | −0.209(4) $cm^{-1}$ | free | -- | -- | -- |
| axial ZFS param. $D$ | #2 | − 235.4(8) $cm^{-1}$ | free | #1 | − 357(2) $cm^{-1}$ | free |
| transverse ZFS param. $E$ | #3 | 6.06(1) $cm^{-1}$ | free | #2 | 6.22(1) $cm^{-1}$ | free |
| isotropic effective $g_{iso}$ parameter | #4 | 1.786(1) | free | #3 | 1.758(1) | free |
| Residual of fit | -- | **0.000018** | | | **0.000025** | |

**Table S4** V-O-coordination distances of $Mg_3V_4(PO_4)_6$ according to previous work[1].

| Vanadium | | | Oxygen | | | V-O-coordination | |
|---|---|---|---|---|---|---|---|
| Label | Ox. | Wyck. | Label | Ox. | Wyckoff | Symmetry | Distance (Å) |
| V1 | 3 | 1a | O1 | -2 | 2i | -x,-y,-z 445 | 2.182 |
| V1 | 3 | 1a | O1 | -2 | 2i | x,y,z 554 | 2.182 |
| V1 | 3 | 1a | O4 | -2 | 2i | -x,-y,-z 455 | 1.978 |
| V1 | 3 | 1a | O4 | -2 | 2i | x,y,z 544 | 1.978 |
| V1 | 3 | 1a | O5 | -2 | 2i | -x,-y,-z 555 | 2.167 |
| V1 | 3 | 1a | O5 | -2 | 2i | x,y,z 444 | 2.167 |
| V2 | 3 | 2i | O1 | -2 | 2i | x,y,z 554 | 2.077 |
| V2 | 3 | 2i | O2 | -2 | 2i | -x,-y,-z 555 | **1.82** |
| V2 | 3 | 2i | O8 | -2 | 2i | -x,-y,-z 555 | 2.04 |
| V2 | 3 | 2i | O9 | -2 | 2i | -x,-y,-z 555 | 2.015 |
| V2 | 3 | 2i | O12 | -2 | 2i | x,y,z 554 | 2.023 |
| V2 | 3 | 2i | O12 | -2 | 2i | -x,-y,-z 555 | 2.041 |
| V3 | 3 | 2i | O3 | -2 | 2i | x,y,z 655 | 1.993 |
| V3 | 3 | 2i | O6 | -2 | 2i | -x,-y,-z 555 | 2.07 |
| V3 | 3 | 2i | O7 | -2 | 2i | -x,-y,-z 555 | 2.025 |
| V3 | 3 | 2i | O7 | -2 | 2i | x,y,z 655 | 2.055 |
| V3 | 3 | 2i | O10 | -2 | 2i | -x,-y,-z 655 | **1.877** |
| V3 | 3 | 2i | O11 | -2 | 2i | -x,-y,-z 555 | 1.926 |

**Magnetic models for DC data fitting:**

$$\hat{H} \;=\; \hat{H}_{EX} + \hat{H}_{CF} + \hat{H}_{ZEE} \qquad \text{(S1)}$$

$\hat{H}_{EX}$: exchange interaction / magnetic coupling

$\hat{H}_{CF}$: crystal field interaction

$\hat{H}_{ZEE}$: Zeeman effect

$$\hat{H}_{EX} = -2 \sum_{i \neq j}^{i,j \in N} \vec{\hat{S}}_i \cdot \overline{\overline{J_{ij}}} \cdot \vec{\hat{S}}_i$$

$\vec{\hat{S}}_i$: Vector operator total spin orbital momentum

$J_{ij}$: complete ((an)isotropic and antisymmetric) exchange tensor

$$\hat{H}_{CF} = \sum_{i=1}^{N} \sum_{k=2,4,6} \sum_{q=-k}^{k} \sigma_i^k B_{ki}^q \theta_k \, \hat{O}_{ki}^q$$

$B_{ki}^q$ : Crystal field parameters ($A_{ki}^q \langle r^k \rangle_i$ in Steven's notation)

$\theta_k$: operator equivalent factors

$\hat{O}_{ki}^q$: operator equivalents

$$\hat{H}_{ZEE} = \mu_B \sum_{i=1}^{N} \left( \sigma_i \vec{\hat{L}}_i \cdot \overline{\overline{I}} + \vec{\hat{S}}_i \cdot \overline{\overline{g_i}} \right) \cdot \vec{B}$$

$\mu_B$: Bohr magneton

$\overline{\overline{I}}$: identity matrix

$\overline{\overline{g_i}}$: $g$-tensor

$\vec{B}$: magnetic induction

## Spin quantum states:

Triplet basis set: $|1,-1>$, $|1,0>$, $|1,1>$

$$G_{m1}(0\ \mathrm{T}): \qquad \frac{1}{\sqrt{2}}|1,1> - \frac{1}{\sqrt{2}}|1,-1> \qquad \text{(S2)}$$

$$G_{m2}(0\ \mathrm{T}): \qquad \frac{1}{\sqrt{2}}|1,1> + \frac{1}{\sqrt{2}}|1,-1> \qquad \text{(S3)}$$

$$H_n: \qquad -|1,0> \qquad \text{(S4)}$$

Determination of relative amount of $V^{2+}$ present in the discharged sample compared to the amount of $V^{3+}$ present in the pristine sample (Langevin paramagnetism):

$$C_{V3+} = \frac{\mu_B^2 \cdot n_{V3+} \cdot \left(\mu_{eff}^{V3+}\right)^2}{3k_B} \qquad \text{(S5)}$$

$$C_{V2+} = \frac{\mu_B^2 \cdot n_{V2+} \cdot \left(\mu_{eff}^{V2+}\right)^2}{3k_B}$$

$$\frac{n_{V2+}}{n_{V3+}} = \frac{C_{V2+} \cdot \left(\mu_{eff}^{V3+}\right)^2}{C_{V3+} \cdot \left(\mu_{eff}^{V2+}\right)^2}$$

$$\frac{n_{V2+}}{n_{V3+}} = \frac{0.1316 \cdot 8}{3.257 \cdot 15} = 0.021549$$

[1] S. H. Porter, J. Xiong, M. Avdeev, D. Merz, P. M. Woodward, Z. Huang, *Inorganic Chemistry* **2016**, 55, 5772.
